# Cryo-EM reveals unique structural features of the FhuCDB *Escherichia coli* ferrichrome importer

**DOI:** 10.1038/s42003-021-02916-2

**Published:** 2021-12-09

**Authors:** Wenxin Hu, Hongjin Zheng

**Affiliations:** Department of Biochemistry and Molecular Genetics, University of Colorado Anschutz Medical Campus, School of Medicine, Aurora, USA

**Keywords:** Cryoelectron microscopy, Membrane proteins

## Abstract

As one of the most elegant biological processes developed in bacteria, the siderophore-mediated iron uptake demands the action of specific ATP-binding cassette (ABC) importers. Although extensive studies have been done on various ABC importers, the molecular basis of these iron-chelated-siderophore importers are still not fully understood. Here, we report the structure of a ferrichrome importer FhuCDB from *Escherichia coli* at 3.4 Å resolution determined by cryo electron microscopy. The structure revealed a monomeric membrane subunit of FhuB with a substrate translocation pathway in the middle. In the pathway, there were unique arrangements of residues, especially layers of methionines. Important residues found in the structure were interrogated by mutagenesis and functional studies. Surprisingly, the importer’s ATPase activity was decreased upon FhuD binding, which deviated from the current understanding about bacterial ABC importers. In summary, to the best of our knowledge, these studies not only reveal a new structural twist in the type II ABC importer subfamily, but also provide biological insights in the transport of iron-chelated siderophores.

## Abstract (web summary)

Wenxin Hu et al. use cryo-EM and biochemical assays to describe the functional activity and structure of the ferrichrome importer, FhuCDB in *E. coli*. Their results provide further insight on the mechanism of siderophore transport in bacteria.

## Introduction

Siderophores are a group of small molecules synthesized and secreted by microorganisms to chelate life-essential iron ions (Fe^3+^) from the environment^1^. The importance of siderophore molecules is further highlighted by the increasing reports of their biological functions other than Fe^3+^ chelating^2^. These functions include binding and transporting a variety of metal ions as metallophores^3,4^, acting as signaling molecules for gene regulation^5^, protecting bacteria from oxidative stress^6,7^, as well as providing antibacterial activity as sideromycins^8,9^. In the extracellular space, secreted siderophores form stable complexes with Fe^3+^ ions, which are recognized and imported by the outer membrane receptors. In the periplasm, a group of soluble proteins named periplasmic substrate-binding proteins (SBP) are generally responsible for binding the siderophore-Fe^3+^ molecules and delivering them to corresponding inner membrane importers. Specifically, in the inner membrane of uropathogenic *Escherichia coli*, there are four such importer systems known so far: YbtPQ importing yersiniabactin^10^, FepBDGC importing enterobactin^11^, FecBCDE importing citrate-based siderophores^12^, as well as FhuCDB importing hydroxamate-based siderophores^13^. Although all of siderophore importers belong to the superfamily of ATP-binding cassette (ABC) transporters, the architecture of the four systems is not the same: YbtPQ resembles the fold of type IV exporter^14^, while the other three seem to be bacterial type II importers.

It is well known that ABC transporters have at least four common domains: two transmembrane domains (TMD) forming a central translocation pathway and two cytosolic nucleotide-binding domains providing necessary energy for the substrate translocation via ATP hydrolysis. For type II ABC importers in gram-negative bacteria, periplasmic substrate-binding proteins (SBP) are usually required to specifically bind and deliver substrates to their TMDs. Available high-resolution structures of type II importers, including vitamin B_12_ importer BtuCDF from *E. coli*^15–18^, heme importers HmuUV from *Y. pestis*^19^ and BhuUVT from *B. cenocepacia*^20^, as well as molybdate importer MolBC from *H. influenzae*^21^, have provided excellent explanation for the general mechanism of substrate import. However, how are different iron-chelated-siderophores imported through related ABC importers is not well understood.

In this study, we functionally characterized the ferrichrome, a hydroxamate-type siderophore molecule, importer FhuCDB complex from *E. coli* and determined its structure at 3.4 Å resolution by single-particle cryo-electron microscopy (cryo-EM). As far as we know, this is the first high-resolution structure of a siderophore importer in the type II ABC importer subfamily. The structure was in the inward-open conformation as the periplasmic side of the transport pathway was closed while the cytoplasmic side was open. The substrate-binding pocket in FhuD (SBP), was fully occupied by loops from FhuB (TMD) as well as several water molecules. In all known type II importer structures, the translocation pathway is formed by homodimers of their membrane subunits. While in FhuCDB, the pathway was in the middle of the FhuB monomer, representing another type of type II importer structure. Along the surface of the pathway, there were small hydrophobic residues, uncharged polar residues, as well as unique layers of Met residues. With the FhuCDB structure and related functional experiments, our study provides important mechanical insights in the import of iron-chelated-siderophores.

## Results and discussion

### ATPase activity and transport assay of the Fhu importer in proteoliposomes

To characterize the function of Fhu importer, we overexpressed the full FhuCDB complex as well as the FhuCB subcomplex (only the membrane and cytosolic components), purified them in detergent lauryl maltose neopentyl glycol (LMNG) (Supplementary Figs. 1 and 2). To perform functional experiments, we incorporated FhuCB into liposomes made of *E. coli* polar extract lipids. The orientation of the reconstituted importers is determined by limited proteolysis of the proteoliposomes using thrombin, followed by western blot using anti-His probe (Supplementary Fig. 3a). The idea was that FhuC from the inside-out importers would be digested and lose its N-terminal His-tag, while FhuC from the right-side-out importers would not be digested. The results (Supplementary Fig. 3b) showed that ~64% FhuC remained intact after the treatment. Considering that the efficiency of the thrombin digestion was ~90%, thus in the proteoliposomes, ~60% of the reconstituted FhuCB were right-side-out, and the rest ~40% of them were inside-out.

**Fig. 1:**
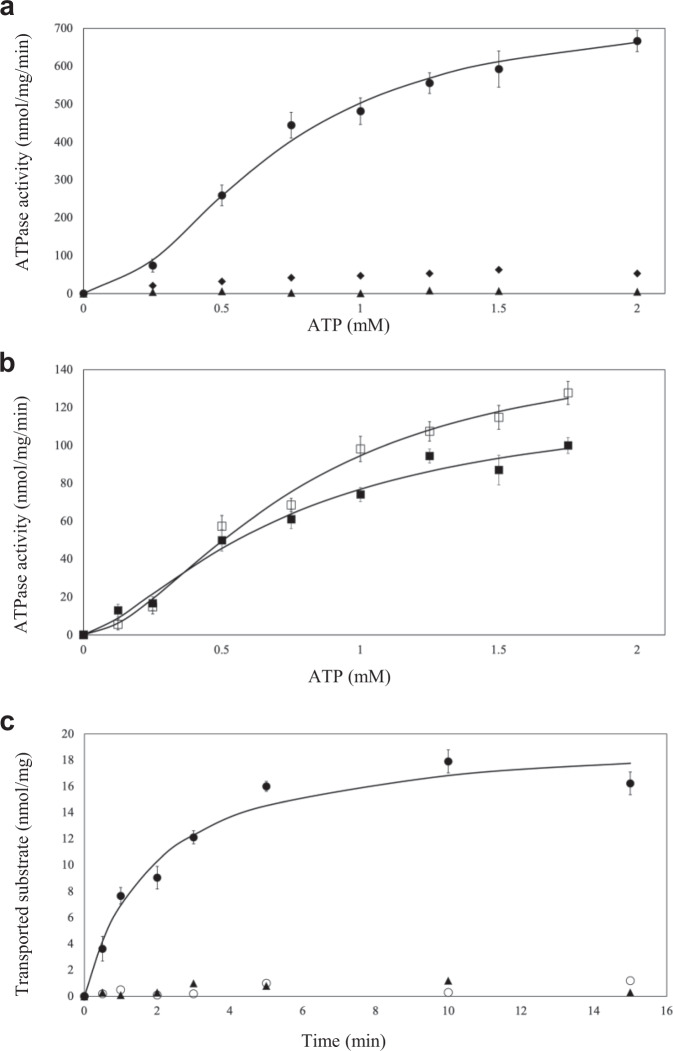
Functional characterization of the Fhu importers in proteoliposomes. **a**, **b** ATPase activity of FhuCB plotted against ATP concentrations. **c** Substrate transport activity of FhuCB in proteoliposomes. The samples are FhuCB (●), vanadate inhibited FhuCB (◆), E173A ATPase null-mutant FhuCB (▲), FhuCB with ferrichrome-loaded FhuD (⎕), FhuCB with FhuD (⎕⎕), and FhuCB transport in the absence of FhuD (○). Error bars are the standard deviation from *n* = 3 independent measurements.

These proteoliposomes were used to measure the ATPase activity of the importers. For the FhuCB subcomplex alone, the initial rate of ATP hydrolysis of the inside-out importers in the first 4 min of reaction was measured and appeared to be linear (Supplementary Fig. 4). We then plotted the initial rates as a function of the ATP concentration (Fig. 1a). Here, the ATPase null-mutant (E173A, in the Walker-B domain of FhuC) was used as a negative control as it had no detectable activity. In addition, vanadate was able to inhibit more than 90% of the ATPase activity. While for the wild-type FhuCB, the data were fit using an expanded version of the Michaelis–Menten equation with Hill coefficient, which yielded the following kinetic constants: V_max_ of ~745 ± 31 nmol/mg/min, K_m_ of ~0.69 ± 0.08 mM, and Hill coefficient of ~1.97 ± 0.08. When comparing to known Type II ABC importers (Supplementary Table 1), we found that FhuCB had a relatively high K_m_ value, suggesting a lower binding affinity to ATP. Furthermore, the sigmoidal shape of the curve with a Hill coefficient of ~1.97 indicated a strong cooperativity between the two ATP binding sites in FhuC. To test if the importer’s ATPase activity was affected by FhuD, we performed the same experiment using FhuCB proteoliposomes with enclosed ferrichrome-loaded FhuD and FhuD only. Surprisingly, the results (Fig. 1b) showed that the ATPase activity of the FhuCB + FhuD was decreased as its kinetic constants were V_max_ of ~127 ± 8 nmol/mg/min, K_m_ of ~0.74 ± 0.05 mM, and Hill coefficient of ~1.45 ± 0.04. While with the ferrichrome-loaded FhuD, the importer’s ATPase activity was also decreased as the kinetic constants were V_max_ of ~155 ± 19 nmol/mg/min, K_m_ of ~0.77 ± 0.06 mM, and Hill coefficient of ~1.73 ± 0.06. The consensus notion in the field is that docking of the SBP to the TMD stimulates the ATPase activity of all known ABC importers^22^. However, in the case of Fhu importer, upon FhuD binding, its ATPase activity was apparently decreased as the V_max_ dropped more than five times. In the meantime, the cooperativity of the ATP binding was weakened, although the ATP binding affinity seemed to be at the same level as indicated by similar K_m_ values. These results suggest that the molecular mechanism of FhuCDB might deviate from the standard model of type II ABC importers derived from BtuCDF.

For the transport assay, an ATP-regenerating system with ATP and MgCl_2_ was included inside the reconstituted proteoliposomes by following the established protocol^23^. The transport activity of the right-side-out FhuCB was quantified by liquid scintillation counting as ^55^Fe-ferrichrome was used as the substrate. The results (Fig. 1c) showed that the ^55^Fe-ferrichrome uptake followed the Michaelis–Menten equation and reached saturation in ~10 min. The initial rate of the transport was ~7.5nmol/mg/min. Ideally, the coupling efficiency of the ATPase activity and transportation rate of ABC transporters is 2, meaning two ATP are hydrolyzed when one substrate is translocated. In the case of FhuCDB, if assuming that FhuCDB is the main species in vivo, the calculated coupling efficiency is ~20, which is higher than the theoretical value of two but reflects the universal characterization of inefficient translocation among most ABC transporters^24^.

### Overall architecture of FhuCDB in the inward-open conformation

**Fig. 2:**
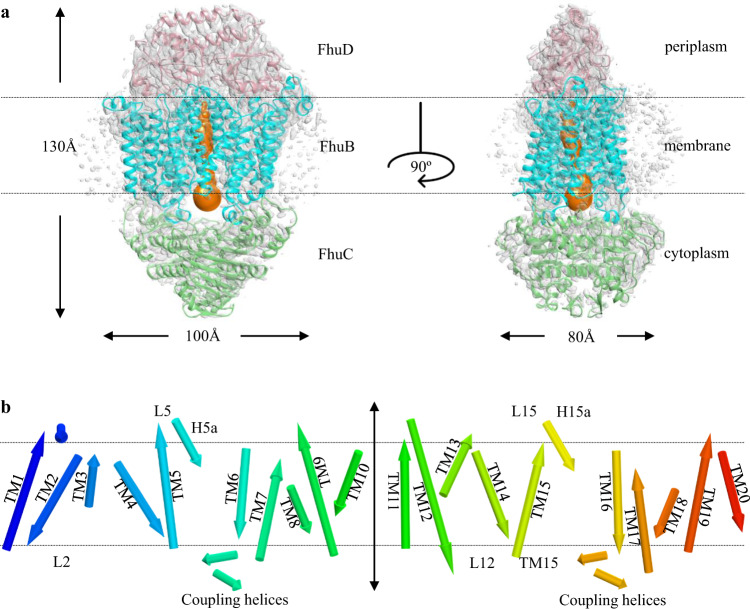
Overall structure of FhuCDB. **a** Atomic model of FhuCDB fits in the cryo-EM density (gray). FhuD in pink, FhuB in cyan, and two FhuC in green. The calculated tunnel in FhuB (orange) indicates that the complex is in the inward-open conformation. **b** Topology of FhuB showing 20 TMs colored in rainbow with N-terminal in blue and C-terminal in red. Important helices and loops discussed in this article, as well as all TMs are labeled.

To understand the molecular basis of this importer, we determined the structure of FhuCDB in LMNG by single-particle cryo-EM at 3.4 Å resolution (Supplementary Table 2, Supplementary Figs. 5–7). The overall size of the complex was ~130 × 100 × 80 Å^3^, with one FhuD on the periplasmic side, one FhuB in the membrane, and two FhuC on the cytosolic side (Fig. 2a). The overall fold of the complex indicated that the importer belonged to the type II ABC importer subfamily. However, a unique feature in FhuCDB was the single membrane subunit of FhuB, comparing to two subunits of homodimers in other known subfamily members. FhuB had 20 transmembrane helices (TM) folding into two domains: N-half with the first ten TMs and C-half with the last ten TMs (Fig. 2b). In between the two halves, there was a central translocation pathway. Based on a tunnel calculation using the MoleOnline server^25^, the FhuB central pathway was open to the cytosolic side and closed to the periplasmic side, indicating an inward-open conformation (Fig. 2a). Right at the cytosolic opening of the central pathway, there was a loop of ~24 residues (residues 324~347) connecting TM10 from N-half and TM11 from C-half that are 45 Å away from each other. Unfortunately, the cryo-EM density of this loop was mostly missing, suggesting that the loop was fairly flexible. Although the loop was unlikely to block the channel opening in this inward-open conformation, it is unclear at this point whether its local state would change in the occluded or outward-open conformations.

### Substrate-binding site in FhuD occupied by FhuB loops and water molecules

**Fig. 3:**
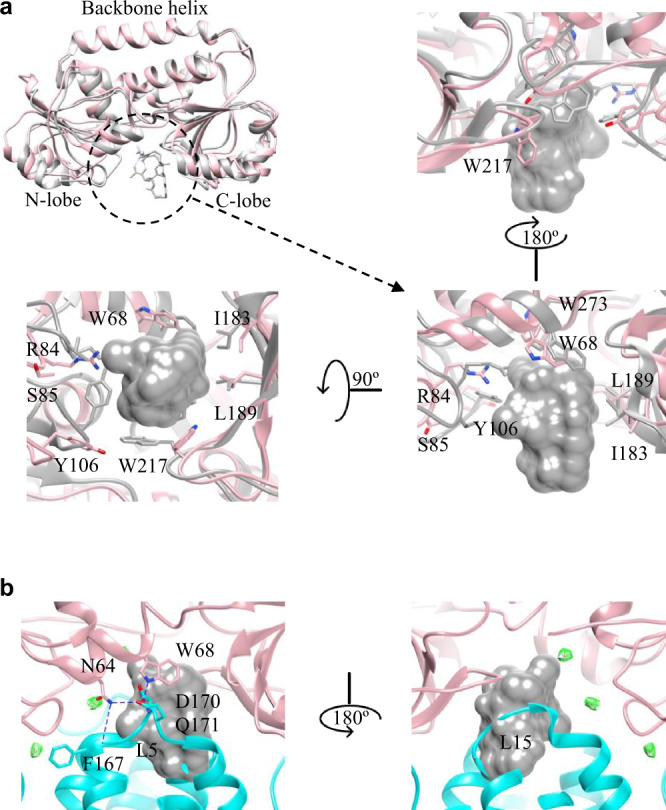
Deformed substrate-binding pocket in FhuD. **a** Superimposed FhuD structures from FhuCDB (pink) and gallichrome-bound FhuD (PDB:1EFD, gray) with substrate-binding residues shown in zoomed-in views. **b** Substrate-binding pocket in FhuD is occupied by two FhuB loops L5 and L15 (cyan) with specific hydrogen bonds (blue). Experimental densities of trapped water molecules are shown in mesh (green).

FhuD is a Cluster A type periplasmic SBP for the FhuCDB import system^26^. It has two lobes (not identical to each other) connected by a hinge α-helix (backbone), which ensures a rigid overall fold and allows only small movement upon substrate binding (Fig. 3a). In our structure, although there was no substrate bound, FhuD could be superimposed well to all previously determined substrate-loaded FhuD structures^27,28^ with a root mean square deviation (r.m.s.d) of 2.3~2.5 Å (Fig. 3a & Supplementary Fig. 8). Using the gallichrome-loaded FhuD (PDB:1EFD) as a comparison, the C-lobe of our FhuD moved marginally with most substrate-interacting residues (hydrophobic I183, L189, and W273) remaining in position except W217, which was flipped 180°. In contrast, the N-lobe moved substantially away from the center, leaving only one residue (W68) unchanged while most other substrate-interacting residues (R84, S85, Y106) pulled away. For instance, the OH group of the Y106 side chain moved as far as ~9 Å. The movement of these residues effectively deformed the original substrate-binding pocket in FhuD and allowed it to be occupied by two loops from FhuB: L5 in between TM5 and H5a as well as L15 in between TM15 and H15a (Fig. 3a). Such loop was originally described in the crystal structure of maltose transporter MalFGK^29^, as the P3 loop from MalG clashed into the maltose-binding site in MBP. After that, similar loops were found in BtuCDF and BhuUVT complex structures^17,20^. Thus, it appears to be a common theme for ABC importers to use these loops to scoop out substrates from their SBP (called “scoop loop”) and subsequently deliver them to their TMD. In FhuCDB, three residues in L5 (F167, D170, Q171) were directly interacting with FhuD residues (N64, W68) via hydrogen bonding. L15 loop protruded into the FhuD pocket without apparent interactions. However, an interesting observation here is that there were three structured water molecules. Specially, two of them were trapped in the interface by the hydrophobic bubble created by L15 and the surrounding FhuD loops. Although such water molecules had been observed in multiple MBP-MalFGK crystal structures, they had never been described in type II importers such as BtuCDF. It is possible that the water molecules are critical for the association of FhuD and FhuB^30^.

### Subunit interactions within FhuCDB

The rearranged FhuD substrate-binding pocket was not the only communication between FhuD and FhuB in the structure. In fact, FhuD docked onto FhuB through a complicated network of interactions. On one hand, the N-lobe of FhuD docked onto the C-half of FhuB via the following specific interactions FhuD-E90/FhuB-R390, FhuD-N88/FhuB-Q636, FhuD-N88/FhuB-Y517, FhuD-E86/FhuB-S515, as well as a hydrogen-bonding network involving a third water molecule (Supplementary Fig. 9a). This water not only connected FhuB-T510 and the backbone of FhuD-T85, but also stabilized the side chain of FhuB-Q507 which in turn interacted with the backbone of FhuB-G107. On the other hand, the C-lobe of FhuD docked onto the N-half of FhuB involving the following FhuD residues H187, S223, D225, R226, and FhuB residues S57, T182, T184, E304 (Supplementary Fig. 9b). Most FhuB residues interacting directly with FhuD were highly conserved (Supplementary Figs. 10–12). Along the central pseudo two-fold axis, we could divide the FhuCDB complex into two halves: one contains FhuD-N-lobe (residues 32–156), FhuB-C-half (residues 348–659), and a FhuC, while the other contains FhuD-C-lobe (residues 157–296), FhuB-N-half (residues 6–323) and the second FhuC. We found that the two FhuD lobes were completely misaligned, the two FhuB halves were superimposed reasonably well, while the two FhuC subunits were almost identical (Supplementary Fig. 13a). The association between FhuB and FhuC was symmetrical: the coupling helices from FhuB-N-half (residues 209~226) matched well with the coupling helices from FhuB-C-half (residues 542~559). Similar to other ABC transporters, the coupling helices docked into the surface grooves of ATPase through mainly hydrophobic interactions and a few hydrogen bonds. It was reported that mutating two conserved Gly residues (G226 and G559) to Ala, Val, or Glu reduced iron hydroxamate uptake^31^. Our structure explained it well, as there was no room for any side chains at this position, thus these mutations would push away the adjacent helix on FhuC and destroy the strong association between the two proteins (Supplementary Fig. 13b).

To further understand the association between subunits, we measured the binding affinity between FhuCB and FhuD in detergent by microscale thermophoresis (MST)^32^. The results (Supplementary Fig. 14, Supplementary Table 3) showed that, without substrate, the binding between FhuCB and FhuD was reasonably tight as the K_d_ values were in the μM range no matter ATP-Mg is present or not. Considering that FhuCDB was overexpressed in rich media, the amount of synthesized siderophore molecules in *E. coli* was negligible. Thus, without in vivo substrates, the naturally strong interaction between FhuCB and FhuD was most likely the reason why we were able to purify FhuCDB as an intact complex. When FhuD was loaded with ferrichrome, the binding was dramatically increased, as the K_d_ values dropped to ~12.2 nM (without ATP-Mg) and ~1.03 nM (with ATP). In addition, we mutated all previously mentioned residues in FhuB (R390, Q636, Q507T510, S515Y517, D170Q171, S57, E304, T182T184) to Ala and found decreased binding affinities between FhuB mutants and FhuD-ferrichrome across the board (Supplementary Table 3). These measurements agreed with a previous report that both FhuD alone and substrate-loaded FhuD were able to interact with FhuB while tested by chemical cross-linking and by prevention of FhuB degradation by added FhuD^33^.

### Potential translocation pathway and the gating

**Fig. 4:**
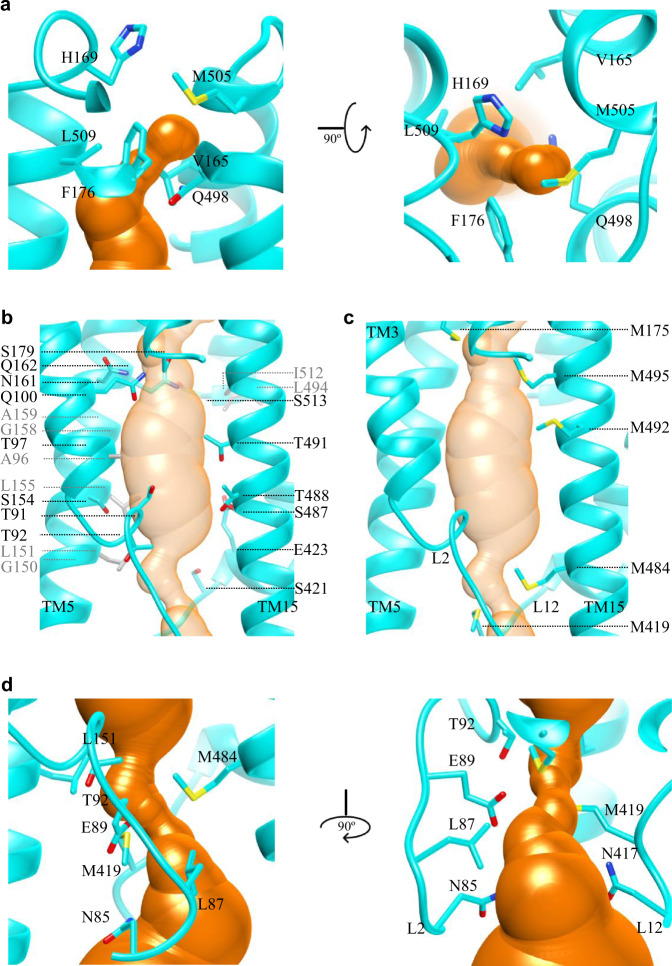
The translocation pathway and its gating in FhuB. **a** The closed periplasmic gate of the translocation pathway (orange) defined by specific residues (cyan). **b**, **c** Three groups of residues along the translocation pathway from three main TMs: 3, 5, and 15. Panel **b** shows two groups: small hydrophobic residues (gray) and polar residues (cyan), while panel **c** shows continuous layers of Met residues (cyan). **d** The narrowest position in the cytoplasmic side of the pathway defined by specific residues.

In all known type II importers, two TMs along the translocation pathway have been extensively discussed: TM5 on one protomer and its counterpart TM5' on the other protomer. Comparing to the structure of inward-open BhuUVT (PDB:5B58), the translocation pathway in FhuB was vividly narrower as TM15 moves ~4.5 Å towards the center while other adjacent TMs stayed at similar positions as in BhuU (Supplementary Fig. 15). The pathway in FhuB was thus unsymmetrically formed by three main TMs: TM3, TM5, and TM15, without TM13 as in BhuUVT (Fig. 4). On the periplasmic side, the pathway was closed by residues from the following regions: TM5 ~ H5a and TM15 ~ H15a. Specifically, these four residues: V165, F176 on the N-half and their counterparts Q498, L509 on the C-half, defined the narrowest position in the pathway, which was then directly closed on top by H169 and M505 (Fig. 4a). Below the periplasmic gate, residues along the path could be grouped into three types: hydrophobic residues with mostly small side chains (A96, G150, L151, L155, G158, A159, L494, I512), polar residues (T91, T92, T97, Q100, S154, N161, Q162, S179, S421, E423, S487, T488, T491, S513) capable of forming hydrogen bonds, as well as Met residues (M175, M495, M492, M484) (Fig. 4c). How do the specific arrangements of residues contribute to the translocation of substrate such as ferrichrome? First, it has been demonstrated that ferrichrome favorably interacts with aromatic rings and thus there are adequate aromatic residues found in the ferrichrome-binding site in FhuD and its outer membrane receptor FhuA^30,34^. While in FhuB, no aromatic residues are found pointing directly towards the central pathway, suggesting that the binding between FhuB and ferrichrome will be rather weak and thus favors rapid translocation. Second, to minimize the energy barrier of transportation, oligosaccharide transporters, such as the maltoporin channel^35^ and Wzm-Wzt transporter^36^, employ a combination of aromatic hydrophobic interactions with a continuous pattern of hydrogen bond donors and acceptors along their translocation pathways. While in the case of FhuCDB, ferrichrome is not inherently hydrophobic, especially its hexapeptide ring. Thus, its translocation should be facilitated by the continuous layers of hydrogen bond donors and acceptors along the way, which are the abundant polar residues especially Thr and Ser. Third, a similar arrangement of Met residues is also observed in another siderophore ABC importer YbtPQ^14^ (Supplementary Fig. 16). To confirm the functional importance of the Met residues along the pathway, we mutated all four of them (M175, M484, M492, M495) to Ala and performed ^55^Fe-ferrichrome transport assay using proteoliposomes. The results (Supplementary Fig. 17) show that the Met mutant FhuCB reconstituted into the liposomes as efficiently as wild-type FhuCB. Although the ATPase activity remains at the similar level, the transport efficiency of the Met to Ala mutant drops ~80%, suggesting potential interactions between the substrate and Met residues. We then mutated all four Met to Leu and found that the transport efficiency drops ~50% comparing to the wild-type FhuB, confirming that the hydrophobic nature of the Met side chains is important for the substrate translocation. However, as the transport efficiency of Met to Ala mutant drops more, these Met residues must provide more than just hydrophobic interactions to the substrate. It is known that the sulfur atom in the Met side chain is generally not active but can occasionally react with some electrophilic centers, even forming hydrogen bonds^37^. Thus, a slight possibility is that in the FhuCDB complex, the Met side chains may transiently attract the chelated Fe^3+^ in ferrichrome using its sulfur atom, which could also contribute to the substrate translocation.

Previous studies on BtuCDF complex revealed two cytoplasmic gates: gate I of the TM5 that was closed in the structure of asymmetrically closed BtuCDF (PDB: 2QI9)^16^, and gate II of the L2 loop between TM2 and TM3 that was closed in the structure of outward-open BtuCD (PDB: 4R9U)^18^. Here, we compared those two structures with our FhuCDB (Supplementary Fig. 18). The comparison showed roughly the same position for the cytosolic half of these TMs: FhuB-TM15 and BtuC-TM5'. However, the positions of TM3, TM5, and L2 in FhuB were drastically different. Specifically, FhuB-TM3 was much closer to the center of the molecule than BtuC-TM3 in both Btu structures, and thus actively participated in the formation of the translocation pathway. As cytoplasmic gate I, the cytoplasmic half of FhuB-TM5 was tilted away from the center, making it different from the closed TM5 in the 2QI9 structure (Supplementary Fig. 18a) but similar to the open TM5 in the 4R9U structure (Supplementary Fig. 18b). Thus, the cytoplasmic gate I was open in our structure. In the meantime, the cytoplasmic gate II in FhuB was also open, because the distance between the L2 and L12 loops were much larger than the closed gate II in the outward-open BtuCD (Supplementary Fig. 18b). The closed gate II in BtuCD was formed by two conserved residues N83 and L85, equivalent to residues N85, N417, and L87, M419 in FhuB, respectively. While in our structure, the narrowest position, which is still open, in the cytoplasmic side of the pathway was defined by residues T92, M484, L151, and E89 (Fig. 4c).

## Conclusions

In this study, we determined the structure of the ferrichrome ABC importer FhuCDB from *E. coli* in the inward-open conformation at 3.4 Å resolution. The structure revealed critical residues regarding subunit interactions, periplasmic gating, as well as transport pathway. The importance of these residues was confirmed by mutagenesis and functional assays. Specifically, along the transport pathway, a continuous layer of Met residues along the transport pathway seemed to be a unique feature of the siderophore ABC importers.

**Fig. 5:**
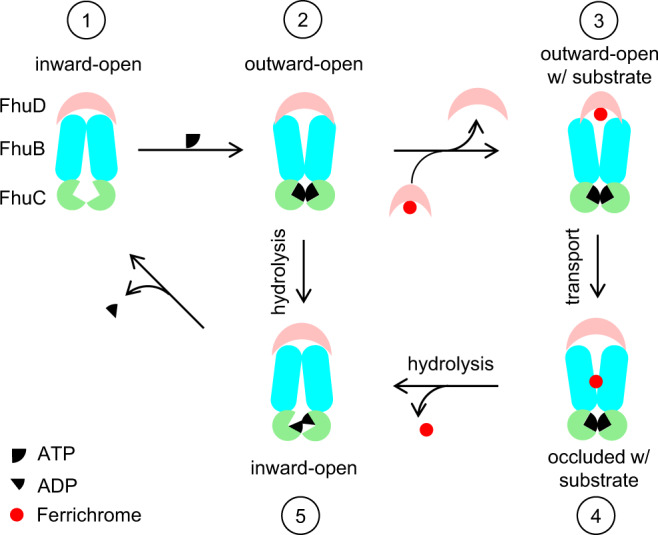
Schematic representation of the transport cycle of FhuCDB. Without the substrate, FhuCDB cycles through inward-open (states 1, 5) and outward-open (state 2) upon ATP binding, ATP hydrolysis, and ADP release. With the substrate, FhuD in FhuCDB (state 2) is replaced by the substrate-loaded FhuD (state 3). The substrate is then released to FhuB and transferred down the translocation pathway (state 4). After ATP hydrolysis, FhuB opens the cytosolic gate and releases the substrate into the cytosol (state 5).

By comparing with other known type II importers, we propose the following molecular mechanism for the substrate import (Fig. 5). First, the FhuCDB structure (state 1) obtained here most likely represent the resting state in the substrate import cycles because of the following three reasons: (1) there is no natural substrate synthesized in *E.coli* growing in rich medium with high iron concentration, and thus no import event happens; (2) FhuD stays on FhuCB despite the natural high ATP concentration in the cells; (3) FhuCDB is on the same operon with overlapping nucleotides between adjacent genes, thus the co-translation of the three proteins should promote the formation of the holo complex. Nevertheless, intracellular ATP could still bind to the resting FhuCDB (state 1) and probably change its conformation to the outward-open (state 2). When there is no substrate present, FhuCDB switches back to inward-open (state 5) after ATP hydrolysis and finally goes back to state 1 after ADP release. States 1, 2, and 5 establish the futile cycle. When we incubated the purified FhuCDB complex at 1 μM with 10 μM ferrichrome and 5 mM ATP-Mg in the solution followed by gel filtration analysis, we found no free FhuD, suggesting that neither ATP nor substrate was enough to disassociate FhuD from FhuCB. Thus, during the futile cycle, FhuD most likely stays associated with FhuCB. Furthermore, when there is substrate present, the only way to start the substrate import seems to be competitively replacing FhuD with substrate-loaded FhuD. This is possible because substrate-loaded FhuD has higher binding affinity to FhuCB as shown in our experiments. It is worth noting that other known Type II ABC importers seem to have an opposite trend of the SBP-TMD association comparing to FhuCDB. For example, in the vitamin B_12_ importer BtuCDF, BtuCD binds to BtuF extremely tight (*K*_d_ = 0.12pM) without the substrate, while binds to substrate-loaded BtuF much weaker (*K*_d_ = 21.1 nM without ATP-Mg and *K*_d_ = 3.6 nM with ATP-Mg)^38^. When FhuD is replaced by substrate-loaded FhuD (state 3), with the action of scoop loops, FhuD opens the pocket and release the substrate into the central translocation pathway in FhuB. Here, ATP should be bound to FhuC in order to change the FhuB conformation to open its periplasmic gate and close its cytoplasmic gates. Then, the substrate is translocated down the pathway via transient interactions with layers of Met and small polar residues (state 4). Last, ATP hydrolysis triggers FhuB to open its cytoplasmic gates and release the substrate into the cytoplasm (states 5 and 1). In other models of Type II ABC importer, a general idea is that SBP dissociates from TMD to prepare the importer for the next cycle of action. In the case of FhuCDB, we argue that FhuCB subcomplex state might be short-lived in vivo, and thus FhuD would not be released from the holo complex in between the import cycles. There are at least three reasons for this argument: (1) the basal ATPase activity of FhuCDB is much lower than FhuCB subcomplex (Fig. 1), suggesting that FhuCDB is conformationally more rigid and is possibly favored in vivo as it consumes less ATP during futile cycles; (2) FhuD could not be separated from FhuCB by incubating with ATP and substrates; (3) in our cryo-EM studies on FhuCDB, we did not find any 2D averages of FhuCB subcomplex alone (without FhuD), indicating low possibility of its physiological existence. Nevertheless, structures of Fhu importers in other conformations and in lipid-surrounded environment determined in the future will provide more insights into its specific molecular mechanism.

## Methods

### Plasmids

For the FhuCDB construct, the whole operon of FhuCDB was cloned from *Escherichia coli* (*E. coli*) genome into a pET15b (Novagen) vector with a His-tag and a thrombin digestion site on the N-termini of FhuC. For the wild-type FhuCB construct, FhuD gene in the middle of the operon was deleted directly from the FhuCDB-pET15b construct. All mutants of FhuCB used in this manuscript were modified on this FhuCB-pET15b construct by direct mutagenesis. For the FhuD construct, the gene was amplified and cloned into a pET15b vector.

### Protein expression and purification

FhuCDB and FhuCB constructs were overexpressed in *E. coli* strain of BL21(DE3) C43 (Sigma-Aldrich) at 18 °C overnight with 0.2 mM Isopropyl β-D-1-thiogalactopyranoside (IPTG, UBPBio). The culture was harvested and resuspended in Buffer A (20 mM HEPES at pH 7.5 and 150 mM NaCl) with 1 mM phenylmethylsulfonyl fluoride (PMSF). The cells were lysed by passing through the M110P microfluidizer (Microfluidics) two times at 15,000 psi. Cell debris was removed by centrifugation at 15,000 g for 30 min. Then, the membrane fractions were collected by ultracentrifugation at 150,000 g for 2 h, resuspended in Buffer A, and stored at −20 °C. To solubilize the complexes in the membrane, 1% lauryl maltose neopentyl glycol (LMNG) was added, and the mixture was incubated at 4 °C for 2 h before ultracentrifugation at 150,000 g for 1 h. Proteins in the supernatant were purified by the affinity chromatography with TALON (Clontech) resin, followed by gel filtration chromatography on a Superose 6 column (GE Healthcare Life Sciences) in Buffer A with 0.001% LMNG. The eluted peak fractions were combined and concentrated to ~5 mg/ml for further experiments, respectively. FhuD was expressed and purified by following a published protocol^28^.

### Reconstitution of FhuCB and FhuCDB into proteoliposomes

The experiment was done by following a previously published protocol^23^. Briefly, 10 mg *E. coli* polar extract lipids (Avanti Polar Lipids) were dissolved in chloroform, dried by nitrogen gas, and immediately resuspended in Buffer A to a final concentration of 10 mg/ml. The large unilamellar liposome vesicles were made by extruding the suspension through a 400 nm polycarbonate membrane filter using a mini extruder (Avanti Polar Lipids). To reconstitute proteoliposomes, the liposomes were destabilized by 0.015% Triton X-100 and then incubated with purified proteins (FhuCDB or FhuCB or FhuCB) at a ratio of 100:1 (wt/wt) for 20 min at room temperature. A total of 100 mg Bio-Beads SM-2 (Bio-Rad Laboratories) was then added to the mixture to absorb all detergents overnight at 4 °C. The reconstituted proteoliposomes were harvested by ultracentrifugation at 180,000 g for 20 min for further experiments.

### Thrombin digestion of the proteoliposomes

To estimate the ratio of inside-out and right-side-out transporters in the proteoliposomes, thrombin digestion was performed. The FhuCDB and FhuCB proteoliposomes were incubated with human α-thrombin (Enzyme Research Laboratories) at a molar ratio of 100:1 at room temperature for 2 h, and then subjected to western blot analysis using HisProbe-HRP conjugate (Thermo Fisher Scientific). The density of the protein bands was quantified using GelAnalyzer 19.1 (www.gelanalyzer.com).

### ATPase activity assay

The ATPase activity was determined using an ATPase/GTPase Activity Assay Kit (Sigma-Aldrich) featuring a detectable fluorescent product from malachite green reacting with released phosphate group. Three proteoliposome samples were used: FhuCB, FhuCB with 10 μM ferrichrome and 1 μM FhuD enclosed, as well as FhuCB with 1 μM FhuD enclosed. 0.002~0.005 mg/ml of these proteoliposomes were incubated with ATP-MgCl_2_ at various concentrations from 0 to 2 mM at 37 °C. Several time points between 0 and 4 min were taken. The amount of phosphate produced by inside-out transporters was detected by absorbance at 620 nm. The data were fitted to the extended Michaelis–Menten equation with Hill coefficient in Excel (Microsoft).

### Transport assay

Proteoliposomes with either FhuCB or FhuCB Met mutants were used, and the transport rate of the right-side-out importers was measured as follows. Briefly, to enclose the ATP-regeneration system, the proteoliposomes, were subjected to three cycles of freeze and thaw with 10 mM ATP, 2 mM MgCl_2_, 0.1 mg/ml pyruvate kinase, and 10 mM phosphoenolpyruvate followed by five times of passing through a 400 nm polycarbonate membrane filter. The proteoliposomes were harvested by ultracentrifugation at 180,000 g for 20 min and then resuspended into Buffer A to a final protein concentration of 0.002 mg/ml. The uptake experiments were carried out at 37 °C with 1.6 μM purified FhuD and 16 μM substrate ^55^Fe-ferrichrome. The reaction was stopped at different time points by adding 200 μl of ice-cold Buffer A, followed by filtration using pre-wetted cellulose nitrate filters. The filters were washed with 1 ml of Buffer A, dried for 1 h, and then dissolved in 3 ml of Filter Count scintillation liquid (Perkin Elmer). The ^55^Fe radioactivity within the proteoliposomes was quantified by using a Tri-carb 2910 TR Scintillation counter (Perkin Elmer).

### Microscale thermophoresis

To measure the binding affinity between FhuD and FhuCB, MST experiments were performed with a Monolith NT.115pico (NanoTemper). A standard protocol was followed^32^. Briefly, the His-tag on purified FhuCB in detergent was labeled with the red-tris-NTA 2^nd^ generation Dye. For each reaction, 40 nM FhuCB was mixed with a serial dilution of 1.6 μM FhuD with substrate ferrichrome or 30 μM FhuD without substrate. Micro thermophoresis was performed using 20% LED power and medium MST power. *K*_d_ values were all calculated using the NanoTemper software with default settings.

### Cryo-EM structure determination and model building

Three microlitre of purified FhuCDB in LMNG at ~1.2 mg/ml were applied to a plasma-cleaned C-flat holy carbon grids (1.2/1.3, 400 mesh, Electron Microscopy Services). The grid was prepared using a Vitrobot Mark IV (Thermo Fisher Scientific) with the environmental chamber set at 100% humidity and 4 °C. The grid was blotted for 3.5 s and then flash frozen in liquid ethane cooled by liquid nitrogen. Cryo-EM data were collected in the Pacific Northwest Cryo-EM Center (PNCC) on a Titan Krios (Thermo Fisher Scientific) operated at 300 keV and equipped with a K3 direct detector (Gatan) together with a Bioquantum energy filter (slit width of 20 eV used). A total of 9142 movies were recorded with a pixel size of 0.399 Å under super-resolution mode, a defocus range of −1–−2.5 μm, and a total dose of ~65 electrons/Å^2^ over 60 frames. The total exposure time was ~2.1 s with a dose rate of ~19.6 electrons/pixel/s. The data were processed using both cryoSPARC v2.15^39^ and Relion 3.1^40^. Specifically, in cryoSPARC, movies were processed with patch motion correction and patch CTF estimation. Approximately 2000 particles were manually picked, and ten class averages were generated by 2D classification. Five good class averages were selected as templates for auto picking. Following standard procedure of particle extraction, 2D classification, ab-initio reconstruction, and heterogeneous refinement, best groups of particles were selected for a final reconstruction at 3.4 Å resolution. The reconstruction and selected particles were then imported into Relion for further 3D classification and 3D auto-refine. The final reconstruction from Relion was at the same resolution as the one from cryoSPARC. In both programs, the gold-standard FSC curves with a 0.143 cutoff were used to determine the resolution. The model building process was carried out in Coot^41^ using the map from Relion. X-ray structure 1ESZ^27^ was used as the initial model to build FhuD, while other part of the FhuCDB model was built manually from the scratch. Briefly, secondary structures were built with the guild from multiple secondary structure prediction programs: Jpred^42^ and PSSpred^43^, as well as the well-known topology of the nucleotide-binding domains. Flexible loops were manually built after placing the secondary structures. The final model was refined in PHENIX^44^. The quality of the model was assessed by MolProbity^45^. Statistical details could be found in Supplementary Table 2. All superpositions of structures were calculated in the program UCSF Chimera with the alignment algorithm of Needleman-Wunsch and BLOSUM-62 matrix^46^.

### Statistics and reproducibility

ATPase activity assays, and substrate transport experiments were independently repeated three times. All the calculations were done in Excel. For Fig. 1, the data were fitted to the extended Michaelis–Menten equation with Hill coefficient. For Supplementary Fig. 4, the data were fit to a linear function.

### Reporting summary

Further information on research design is available in the Nature Research Reporting Summary linked to this article.

## Supplementary information

Supplementary Information

Description of Additional Supplementary Files

Supplementary Data 1

Reporting Summary

## Data Availability

Cryo-EM map of FhuCDB was deposited in the Electron Microscopy Data Bank under the accession code EMD-23251. Coordinates of the atomic model of FhuCDB was deposited in the Protein Data Bank under the accession code 7LB8. Uncropped SDS-PAGE and Western blots for Supplementary Figs. 2b, 3b and 17a are provided in Supplementary Fig. 19. Data for Fig. 1 are provided in Supplementary Data 1. All other data are available from the corresponding author upon reasonable request.
