# Author Correction: Cryo-EM reveals unique structural features of the FhuCDB *Escherichia coli* ferrichrome importer

**DOI:** 10.1038/s42003-021-02979-1

**Published:** 2022-01-04

**Authors:** Wenxin Hu, Hongjin Zheng

**Affiliations:** Department of Biochemistry and Molecular Genetics, University of Colorado Anschutz Medical Campus, School of Medicine, Aurora, CO USA

**Keywords:** Cryoelectron microscopy, Membrane proteins

Correction to: *Communications Biology* 10.1038/s42003-021-02916-2, published online 09 December 2021.

In the Acknowledgements section, the sentence ‘(supported by NIH Grant U24GM129547)’ should have read ‘A portion of this research was supported by NIH grant U24GM129547 and performed at the PNCC at OHSU and accessed through EMSL (grid.436923.9), a DOE Office of Science User Facility sponsored by the Office of Biological and Environmental Research.’. The original article has been corrected.

