# Supplementary material for: Cryo-EM reveals unique structural features of the FhuCDB Escherichia coli ferrichrome importer
Source: Commun Biol. 2021 Dec 9;4:1383. doi: 10.1038/s42003-021-02916-2 (PMC8660799; doi:10.1038/s42003-021-02916-2)
Supplement: Supplementary file 1 — Supplementary Information [file 42003_2021_2916_MOESM1_ESM.pdf]

Supplementary Figure 1. Schematic representation of the siderophore mediated iron uptake process in bacteria. The process can be briefly described in the following six steps. 1) siderophores (such as ferrichrome) are synthesized in the cytosol and secreted out; 2) In the extracellular space, siderophores form stable complexes with Fe^{3+}: 3) siderophore-Fe^{3+} complexes are recognized and transported into the periplasm by TonB-dependent beta-barrel outer membrane receptors; 4) In the periplasm, siderophore-Fe^{3+} molecules are usually recognized by corresponding soluble SBPs (such as FhuD), and then delivered to their specific inner membrane importer (such as FhuCB); 5) In the inner membrane, the importers go through a series of conformational changes to actively import the substrate. These actions are powered by the cytosolic ATP hydrolysis; 6) In the cytoplasm, Fe^{3+} is reduced to Fe^{2+} and released from the siderophores to participate in various biological processes.

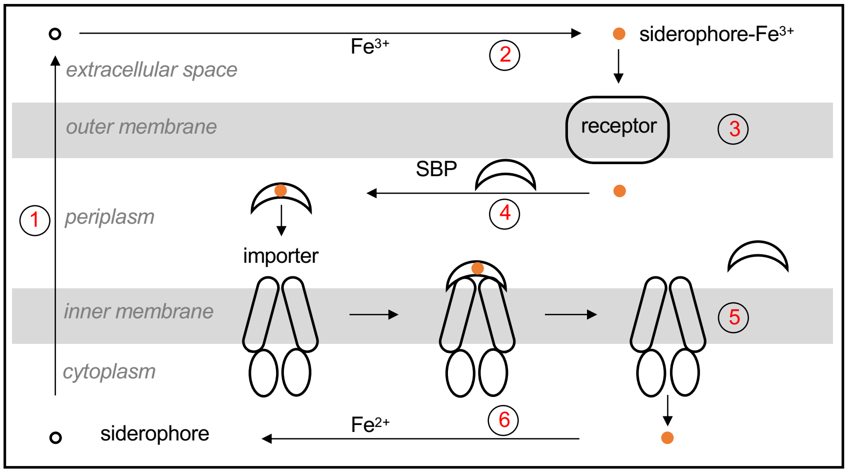

Supplementary Figure 2. Purification of the Fhu importer. a, Gel filtration profiles of the purified FhuCB (orange) and FhuCDB (blue) in detergent LMNG on a S6 column. **b,** SDS-PAGE of the purified FhuCDB (lane 1), FhuCB (lane 3) and FhuD (lane 4). The molecular weight ladder is shown in lane 2.

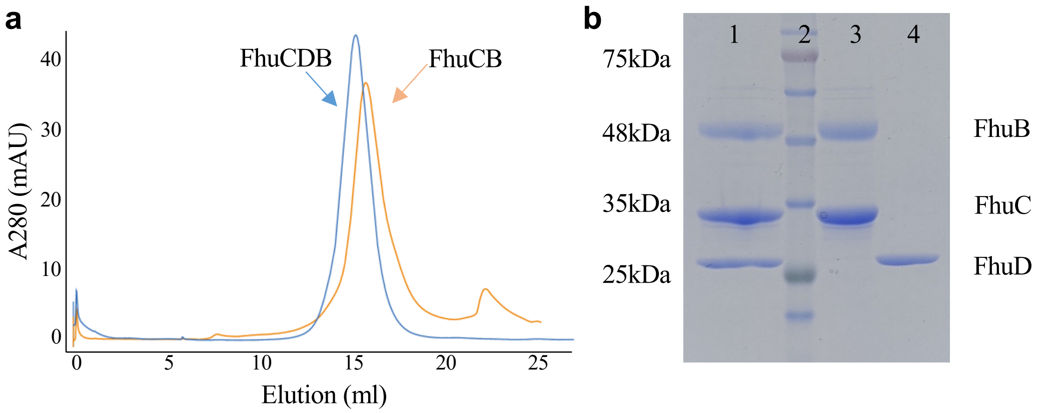

Supplementary Figure 3. Orientational analysis of the reconstituted FhuCB. a, A cartoon representation of a proteoliposome (gray) with two possible orientations of the importers. **b**, Western blot of the thrombin-treated proteoliposomes. Lane 1: untreated FhuCB; lane 2: treated FhuCB in detergent: lane 3: treated FhuCB.

a

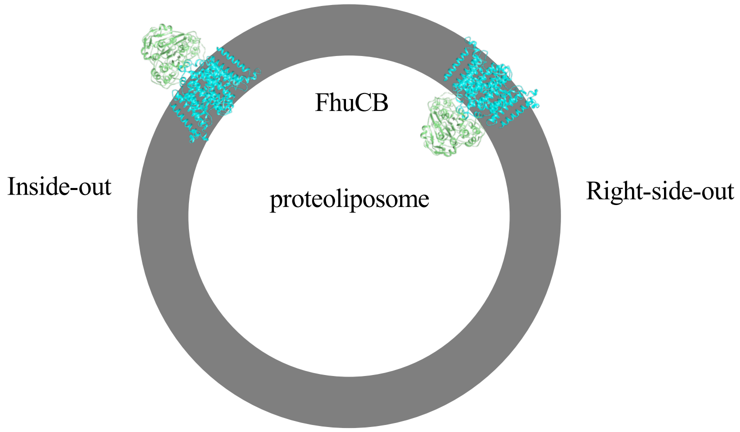

b

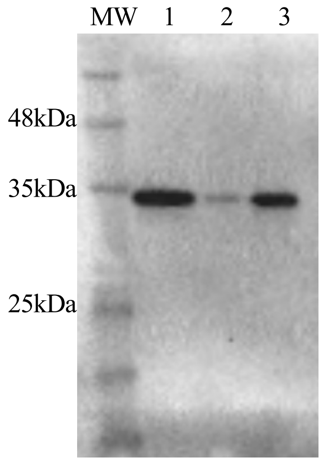

Supplementary Figure 4 ATPase activity of the FhuCB in proteoliposomes. The initial ATP hydrolysis rates of FhuCB over the first 4 min of reaction at different ATP concentrations of 2mM (blue), 1mM (orange) and 0.5mM (grey) . The calculated R^2 values of each linear fit are shown.

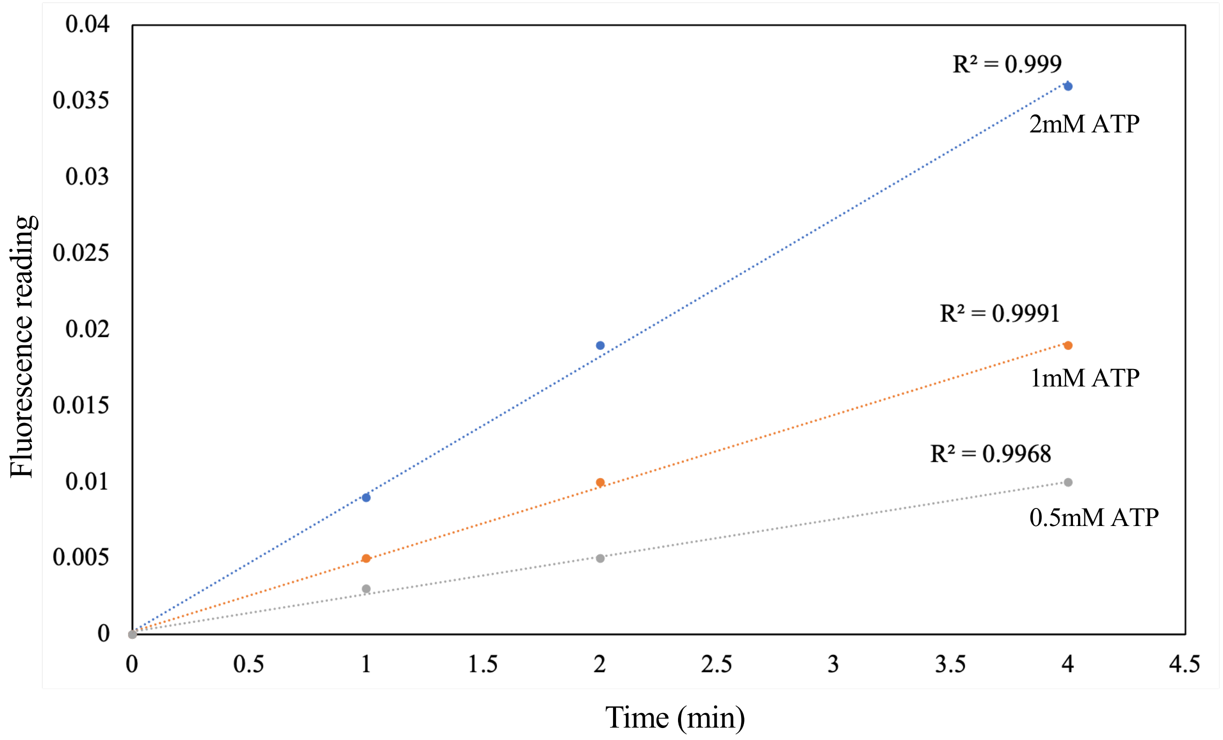

Supplementary Figure 5. cryo-EM analysis of FhuCDB. a, A representative cryo-EM image of FhuCDB. **b,** 2D class averages of FhuCDB. **c**, the final reconstruction of FhuCDB colored by local resolution estimation calculated by Relion. **d**, 3.4Å resolution of the FhuCDB final reconstruction indicated by the gold-standard FSC curve.

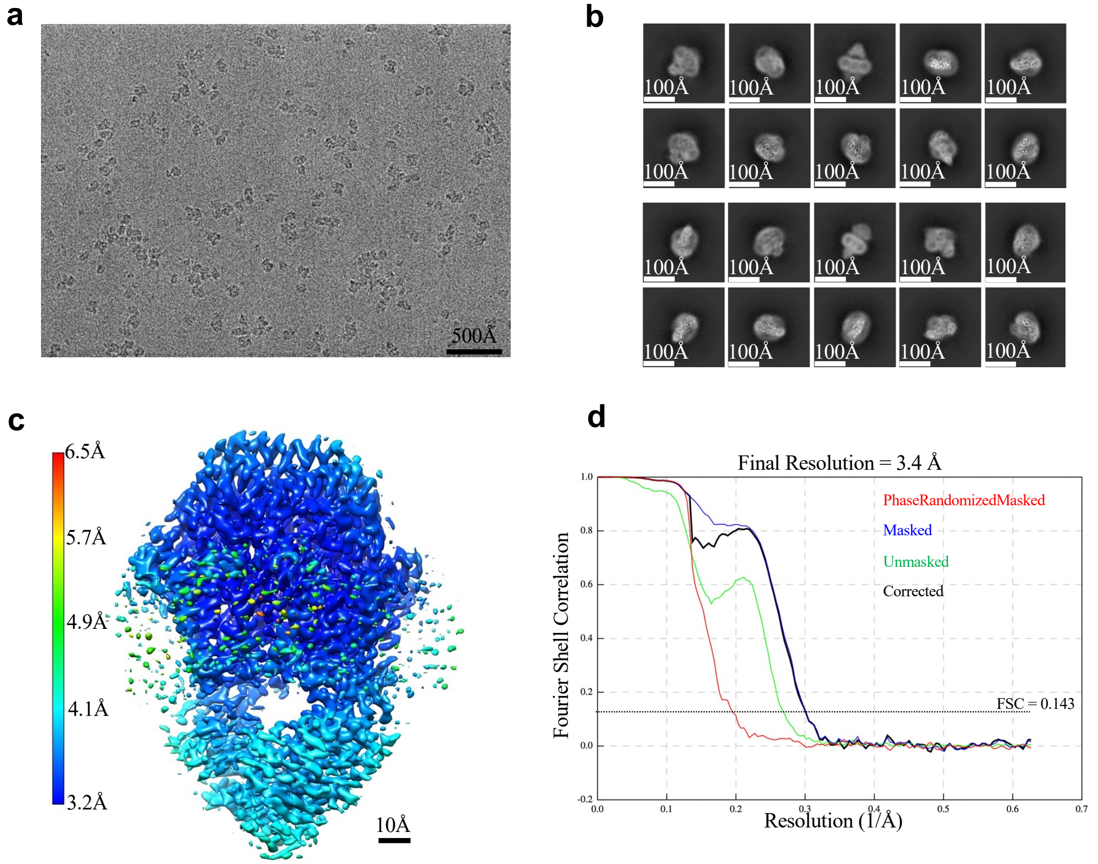

Supplementary Figure 6. Cryo-EM data processing workflow. The data were processed in both cryoSPARC and Relion software packages. Final reconstructions from both software are at the same 3.4Å resolution.

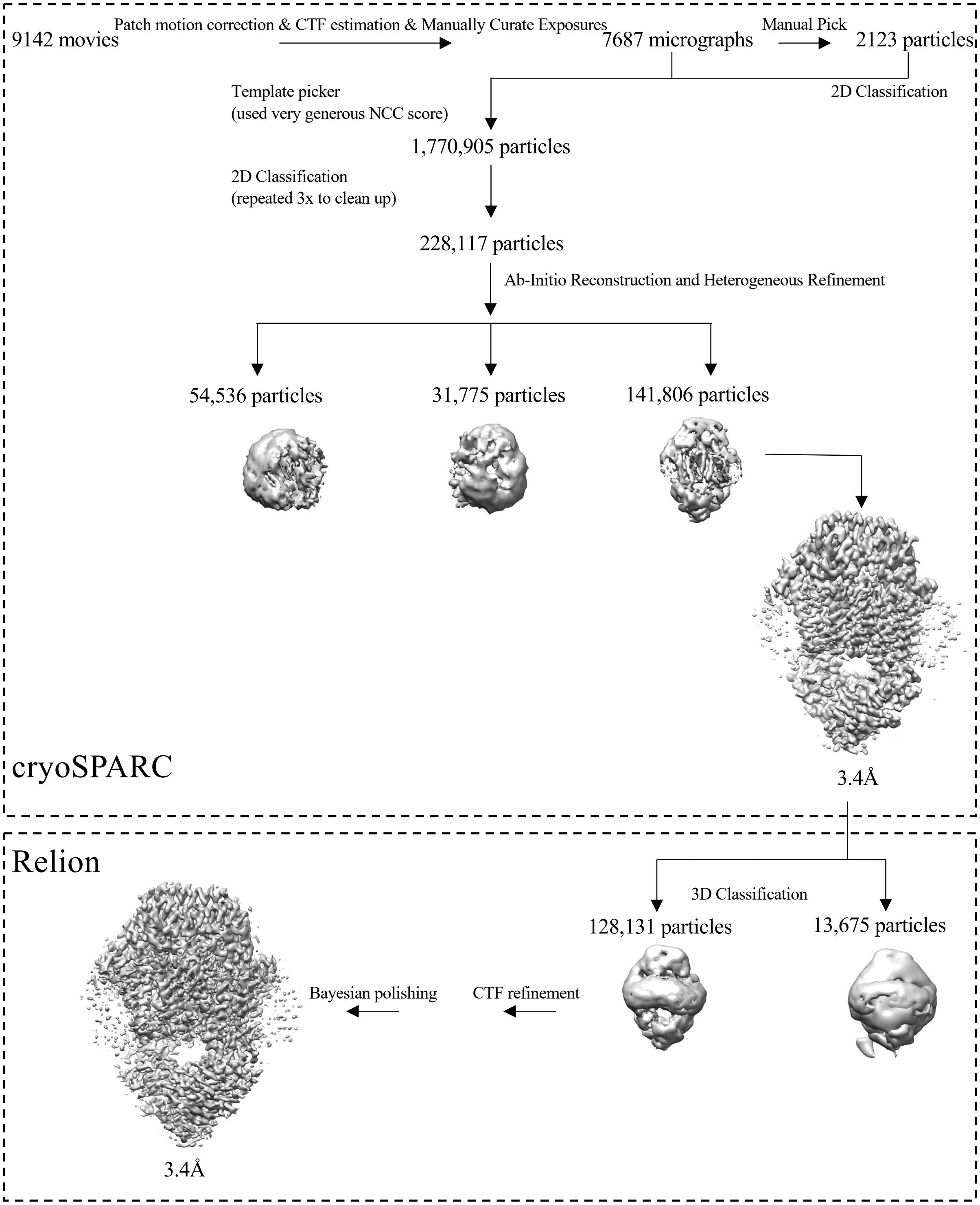

Supplementary Figure 7. FhuCDB model fits in the experimental density. To demonstrate the quality of the cryo-EM map, part of the atomic model of FhuCDB is overlayed with the density. These include all 20 TMs from FhuB (cyan sticks, with starting and ending residues labeled), as well as the backbone helix and the β-sheet with 5 β-strand of FhuD (pink sticks).

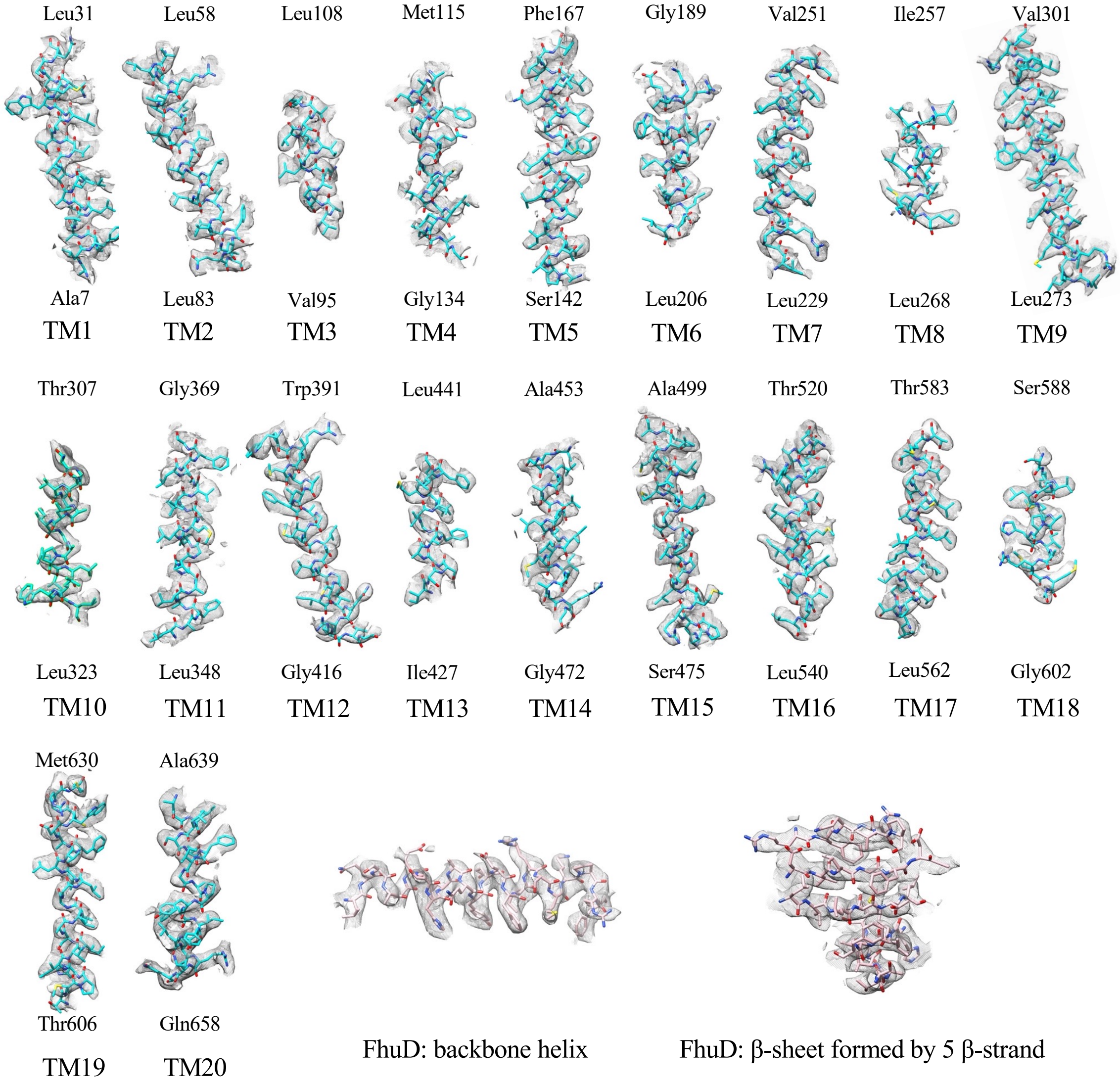

Supplementary Figure 8. Superposition of FhuD structures. FhuD in the FhuCDB complex (pink) is superimposed with **a**) coprogen-bound FhuD from PDB:1ESZ (Clarke et al., 27), **b**) albomycin-delta2-bound FhuD from PDB:1K7S (Clarke et al., 28), and **c**) desferal-bound FhuD from PDB:1K2V (Clarke et al., 28). All the substrate-bound FhuD are colored in gray with the substrate shown as sticks.

a

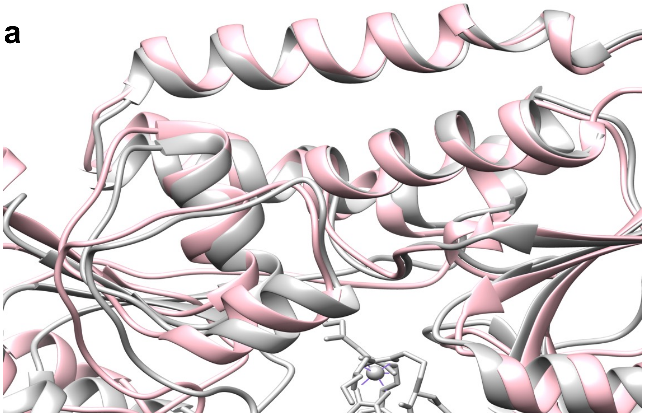

b

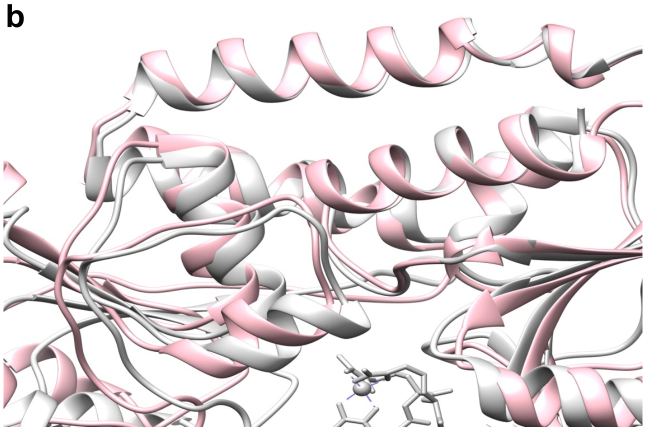

c

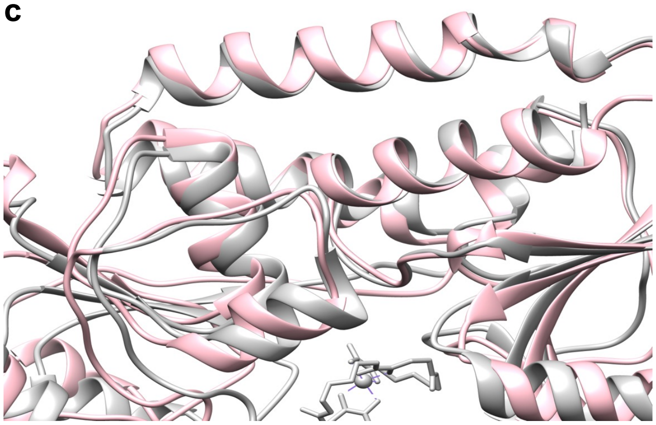

Supplementary Figure 9. Interactions between FhuD and FhuB. a, Specific interactions between FhuD-C-lobe and FhuB-N-half. **b**, Specific interactions between FhuD-N-lobe and FhuB-C-half.

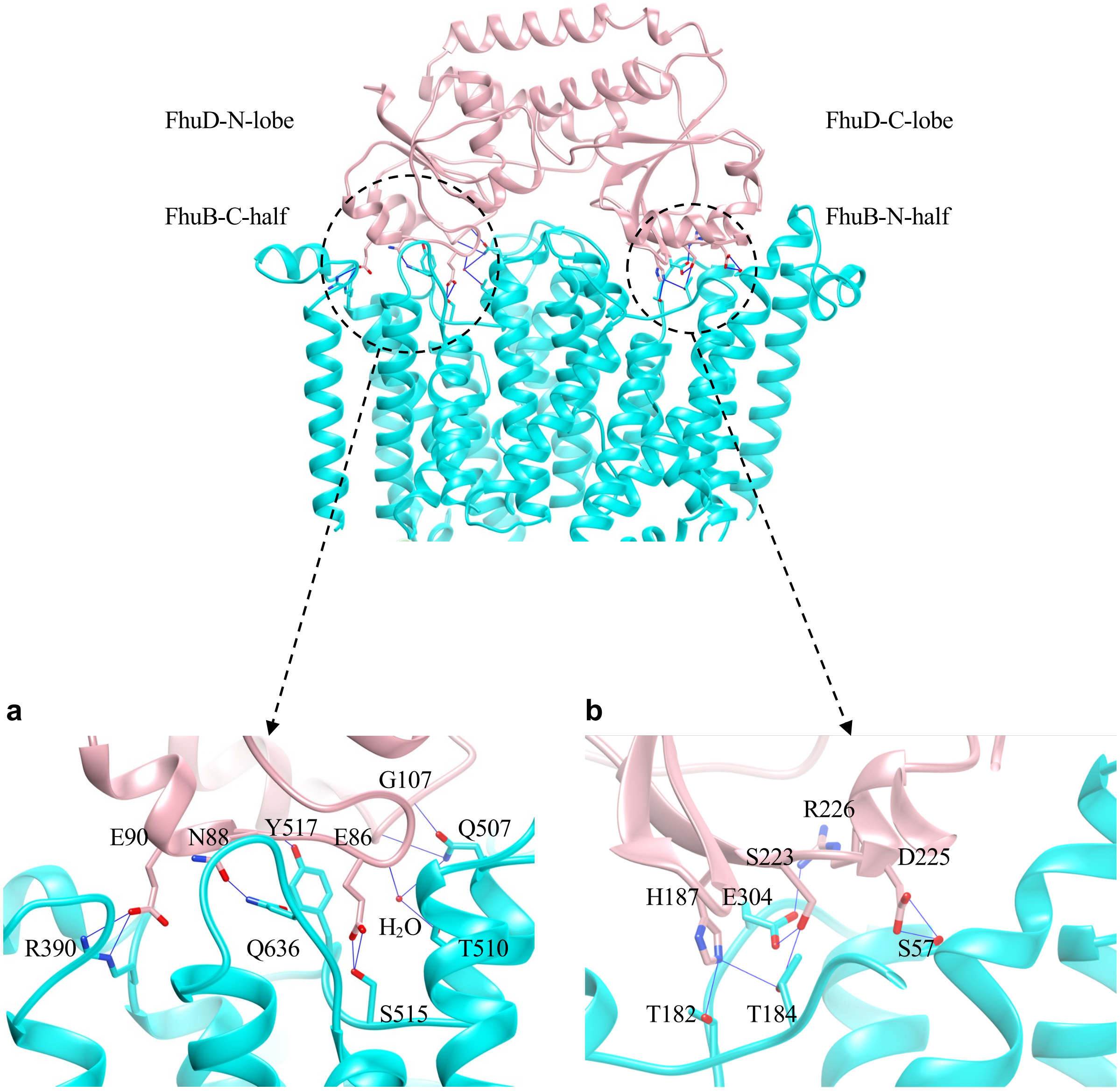

Supplementary Figure 10. Multiple sequence alignment of FhuB from different species. The strains used and their sequence identity to *E. coli* FhuB are: *Salmonella bongori* (88.8%), *Atlantibacter subterranean* (80.5%), *Mixta gaviniae* (70.0%), *Xenorhabdus miraniensis* (60.2%), *Paraburkholderia fungorum* (49.7%), *Phyllobacterium sophorae (*40.7%), *Vibrio rotiferianus (*36.4%), *Oceanobacillus damuensis* (32.8%), *Sediminibacillus albus* (34.6%). Residues involved in FhuD-FhuB interaction are colored in yellow. Residues along the translocation pathway are colored in gray (small hydrophobic), red (polar) and blue (Mets).

```
                                     TM1                             TM2
Ec 1   ------------MSKRIA------LFPA--LLLALLVIVATALTWMNFSQALPRSQWAQAAWSPDIDVIEQMIFHYSLLP  60
Sb 1   ------------MSKRIA------RLPA--FLLVGLFVIAAWLTWTNLSVALPRDQWRQAMWSPSVDAIGQMVFHYSLLP  60
As 1   ------------MRAKSN------LFAP--VLLSVMFLAGLWLTWFNLNHQLPRDQWSAALWHPSQDALDQMLFHYSALP  60
Mg 1   ------------MR-RSW------TFPA--TLLGALFLVALALSGLNLTHHLPQRQWLQALWQPDRHDIDQMVFHFSLLP  59
Xm 1   ------------MR-RLWAVPLLLLFPA--LLL----------TLYNLQQQLPVLQWWQSLHSPDIGNIRQIVVNYSMLP  55
Pf 1   MNFARPIRPIRPLRPDARRFTLRAWLPA--AIGLLALGAALALSVDSLSAILPASQWWHALWSPDTKDIQQIIAHDSWLP  78
Ps 1   -------------MKSLH--PHPLFWAG------LLSCVALAITALNLGPMISIIGWRAAFSAPSPSDSLAVLVHYSLLP  59
Vr 1   ------------MR----------AFPLS--LVILLSLFTMSVGALHLSQTTAFSIGFASLWQPDFQSINSVLLHFSWWP  56
Od 1   ---------MDTMLRK-W-IKAVLTFGGGTALLCILTFVHINQGSVSISFASVVD----AIFVPQ-DLLEHHTVRYLRMP  64
Sa 1   ---------MINVFRSIW-IKTALTFGGGAVLLVALLVVHINQGSVELPLHVVAN----ALFAPE-DIIEHHTVRFLRLP  65

              TM2                         TM3                 TM4
61   RLAISLLVGAGLGLVGVLFQQVLRNPLAEPTTLGVATGAQLGITVTTLWAIPGA-MASQFA----AQAGACVVGLIVFGV  135
61   RLAISLLVGAGLGLVGVLFQQVLRNPLAEPTTLGVATGAQLGITVTTLWAIPGA-LATQFA----ALTGACIVGALVFGV  135
61   RLALSLLVGAGLGLVGVLFQQVLRNPLAEPTTLGVATGAQLGVAIATLWALPGGYMTSQFA----ALAGSALVGLIVFGV  136
60   RLAIALLAGAGLGLVGVLFQQILRNPLAEPTTLGVASGAQLGLTVVTLWALPGGQMTQQIA----AMTGAVLIGLLVFGI  135
56   RLAISLLIGAGLGLAGLLFQQVLRNPLAEPATLGVSAGAQCGMTVALLLAIPSDSLLMQLA----ALAGSVTVALVVFGL  131
79   RLVISWLCGATLGLAGTIFQQVLRNPLAEPMTLGVSAGAQLALTVATLWAPALLG-ADQWI----ALGGALITAAIVFGL  153
60   RLAMACVCGFALGLSGMLFQHVLRNPLASPSTLGVEAGAQLALALAMLWFPALL----GWSRDATTALGGFAAMALVFAA  135
57   RVFTTVISGAGLAVAGVLMQQVLRNPLASPTTLGVASGANFTLMLATLFTPWML----DFSANLVALVGGVTSMGLVMLL  132
65   RAMMGIIAGGALAVSGVILQTVTKNPLASASTLGIHSGTYFAVVLSTVLFPQAL----VGNGIIVAFSGGILTFLTVFLL  140
66   RAVIGILAGGALAVSGVILQTITKNPLASASTLGIHAGTYFAVVFSTIFIPAMI----PGQGLIVAFAGGILTALLVYGL  141

                     TM5                H5a              TM6
136  AWGKRLSPVTLILAGLVVSLYCGAINQLLVIFHHDQLQSMFLWSTGTLTQTDWGGVERLWPQLLGGVMLTLLLLRPLTLM  215
136  AWGKRLSPVTLILAGLVVSLYCGAINQLLVIFHHDQLQSMFLWSTGTLTQTDWSGVQRLWPQLVGGVMLTLLLLRPMTLM  215
137  AWNKRLSPITLILAGLVVSLYCGAVNQLIVIFHHDQLQSVFLWSTGALNQTDWSIVSRLWPQLLGGVMLTLLLLRPLTLM  216
136  AWGKRLSPVTLILAGLVMSLYCGAVNQIFAIFKHDQLQNLFLWSTGSLSQQDWSNVTFLLPRLLAGFVLALLLRRPLTLL  215
132  SWGRRLSPVTLILAGLVMGLYCGAVNSLLALFNYEKLQGMFIWASGSLVQHDWQIVLWMLPILLISLLLTLLLLRPLTLL  211
154  SWRSGLAPVTVAVAGLVVTLYCGSIGVALQLFYAPYLRGLFIWGGGSLVQQDWSNVTMLASRLAIGGVLAFLLLRPMTLF  233
136  AWRLKFQPVAIILSGLVVGLYCTAIVTLLTLMNDHYLSGLFIWGGGSLNQNDWRQVQGLFPKVVACSLVAFMLARQLSVL  215
133  SWRRALSPTVVVVSGLVINLYFASLSTVILMMNTEKLNGLMIWGAGSLVQSGWDDFSYLAPRVAIVALVAFLFAKPLSLL  212
141  SGGNKATPVRMVLAGMIVTLLFSSLTSVLQIFYENETAGLFLWGSGTLVQNDWSGVQFSLPIVVAGLIVILLMSPKLDTL  220
142  AGGAAANPVKMVLAGMVVTIMFSSFTSVLQIFYENETAGLFLWGAGTLVQNDWGGVQFSLPFIIVGLCGVFFIASQLDIF  221
```

Supplementary Figure 11. Multiple sequence alignment of FhuB from different species. The strains used and their sequence identity to *E. coli* FhuB are: *Salmonella bongori* (88.8%), *Atlantibacter subterranean* (80.5%), *Mixta gaviniae* (70.0%), *Xenorhabdus miraniensis* (60.2%), *Paraburkholderia fungorum* (49.7%), *Phyllobacterium sophorae (*40.7%), *Vibrio rotiferianus (*36.4%), *Oceanobacillus damuensis* (32.8%), *Sediminibacillus albus* (34.6%). Residues involved in FhuD-FhuB interaction are colored in yellow. Residues along the translocation pathway are colored in gray (small hydrophobic), red (polar) and blue (Mets).

```
                            TM7                      TM8              TM9
Ec 216  GLDDGVARNLGLALSLARLAALSLAIVISALLVNAVGIIGFIGLFAPLLAKMLGARRLLPRLMLASLIGALILWLSDQII  295
Sb 216  GLDDGVARNLGLALSLARLAVLSLAIVLSALLVNAVGIIGFIGLFAPLLAKMLGARRLLARLMLAPLIGALILWLSDQII  295
As 217  GLDDGVARNLGLALSMARLAALVLAIAISALLVNAVGIIGFIGLFAPLLAKMLGARRLLSRLILAPLIGALILWLSDQMI  296
Mg 216  GLDDGVARNLGMALSLVRLAALGLAIILSAMLVNAVGIIAFIALFSPLMAKLLGARRLFSRMLLAPLIGALLLWLADQCV  295
Xm 212  GLDDVVAKNLGLGLNSARLVALSLAIVLSALMVNAVGVIGFIGLFAPLLARMCGARRLEQQVLMAPLIGALLLWLTDQVV  291
Pf 234  GLDDVNARSLGMSLRRMRLAALALAVVLSAAVVSAVGVIGFVGLAAPALAQMAGARRLSQRLVIAPLCGALILWLTDQAV  313
Ps 216  TLGD-TAQSLGIKLQFTRAGVLFVAVALTAFTVSAVGVIGFLGLAAPAIARATGARRIKSRMIVSPLIGSLLLVIIDQVL  294
Vr 213  ELSEQGAKSLGVSLAKLRFVCLGLAVLMTSWVVATVGIIGFIGLAAPAITRLIGVHKIQHKLWVSMLIGAMLLTLTDLVI  292
Od 221  TLGDDIAVALGQKVSLVKLVAILAAVLLTSVTVSIVGPIGFVGLIAPHIIKLIGYRNHLPLIIASFIWGANVLVFADILA  300
Sa 222  RLGEDVASSLGQNVGRLRLISLLIAVFLTAVTVSVVGPIGFVGLIAPHLLKLIGFRSHYLLVAGSFIWGANVLLGADVLA  301

                     TM10                                                TM11
   296  LWLTRVW-MEVSTGSVTALIGAPLLLWLLPRL-RSISAP--DMKVNDRVAAERQHVL-------AFALAG-GVLLLMAVV  363
   296  LWLTRVW-MEVSTGSVTAFIGAPLLLWLLPRL-KSMSAP--DMNTSDRVAAERRHVL-------AFAVAG-GALLLLATL  363
   297  VWLTRVW-MEVSTGAVTALIGAPLLLWLLPRL-RTQSAP--SLDAGDHIPVERQHVL-------LFSLAG-VGVLLLMIA  364
   296  IWLADNW-REISTGTLTALTGAPILLWLLPRL-RTGAVPP-QLNQGDSVPAERQHVL-------LWLLAALGVLLLLAT-  364
   292  QWLATVW-RSIPTGSAVALIGAPILLWLLPHL-RTYGQRP-TFPVAEQTIIERRALL-------RWFFFG-ILLLSVLII  360
   314  QQLSGVMGDLIPTGAATALFGAPLMLMLLARMHRATRLPDGNDNAARQHPVAAASLR-------RRLISV-VVITLVALV  385
   295  QIYAAKTSVHLPTGAITALIGVPVLLIVILRRGRSMLAAA-----DGKAALPASRLP-------LRVIASIALLLLVVAS  362
   293  QQLPGLMASFIPTGAATAALGAPLLLWLLPRLSQKTQGHTQTVLTRHSERVNKLNKH-----------AIAFTLLIILAA  361
   301  RIID-PSFSELPVGSITALIGAPWLIYLVLRMKKRKYGEENSSVLAGKISIGMPLKIVVPVLIAVILITLLISLASGNNG  379
   302  RIID-TSFSELPVGSITALIGAPWLIWLVLNKRNRQYGDKQTGVLAGSVRLPFSSRLLLPILVILLAGAIFIGLTAGNYG  380

                                    TM12                                    TM13
   364  VALSFGRDAHGWTWASGALLED--LMPWRWPRIMAALFAGVMLAVAGCIIQRLTGNPMASPEVLGISSGAAFGVVLMLFL  441
   364  VALSFGRDAHGWTWASGTLLEE--LMPWRWPRILVALMAGVMLAVAGCIIQRLTGNPMASPEVLGISSGAAFGVVLMLFL  441
   365  VALTFGRDAHGWTWASGDLLRD--LLPWRWPRVLAAMTAGCMLAVAGCIIQRLTGNAMASPEVLGISSGAAFGVVLMLFL  442
   365  MALSLGRDAQGWQWASGELWHQ--LQPWRAPRMAAALAAGVMLGVAGSVIQRLTGNPMASPEVLGISSGAACGVVLMLFL  442
   361  AALLFGRDSEGWHWAQGEQFAE--LLPWRWPRVIAALCAGMMLAVAGTLIQKLTGNPMASPEVLGITSGAGLGVLTLLLM  438
   386  LALDFGHGPAGWHFSSLDEFSR--VAIWRAPRALAALAAGAMLAVAGTLMQRVTGNPLASPEVLGVSAGAALGLIAMVVG  463
   363  IAVFVGRDVDGAWISQGGATLES-LLPWRGPRLVAAMSAGLMLALAGSLLQRLTGNSMASPEVLGVSAGTAFGQLCILFI  441
   362  VVLFSTVSVQNQGWTSLFSTHEWLFLEWRTPRLLAAAMAGAMLAIAGTMIQRLSGNPMASPEVIGISSGTAMGLIIAMFT  441
   380  ---FEPIVAYQAFFGNGNEFMKNLILELRFPRSLVALLSGMLLAISGLIFQGVLRNPLADPSVIGITSGAGVGALLMMYV  456
   381  ---IQIADSISAVMGNGNEFLRNMIVNLRLPRLLVAALSGALLAVSGFVFQGVLRNPLADPSVIGITSGAGVGALLVIFS  457
```

Supplementary Figure 12. Multiple sequence alignment of FhuB from different species. The strains used and their sequence identity to *E. coli* FhuB are: *Salmonella bongori* (88.8%), *Atlantibacter subterranean* (80.5%), *Mixta gaviniae* (70.0%), *Xenorhabdus miraniensis* (60.2%), *Paraburkholderia fungorum* (49.7%), *Phyllobacterium sophorae (*40.7%), *Vibrio rotiferianus (*36.4%), *Oceanobacillus damuensis* (32.8%), *Sediminibacillus albus* (34.6%). Residues involved in FhuD-FhuB interaction are colored in yellow. Residues along the translocation pathway are colored in gray (small hydrophobic), red (polar) and blue (Mets).

```
                                 TM14                    TM15                    H15a
Ec 442  VPGNAFGWLLPAGS-LGAAVTLLIIMIAAGRGGFSPHRMLLAGMALSTAFTMLLMMLQASGDPRMAQVLTWISGSTYNAT  520
Sb 442  VPGNAFGWLLPAGS-LGAAATLLIIMIAAGRGGFSPQRMLLAGMALSTAFTMLLMMLQASGDPRMAEVLTWISGSTYNAT  520
As 443  VPGDAFGWLLPAGS-LGAAATLLIIMIAAGRGGFSPHRMLLAGMALSTAFTMLLMMLQASGDPRMAKVITWISGSTYSVT  521
Mg 443  VPGDAFGWLLPAGS-LGAAVTLLLIMMVAGRGGFSPERMLLAGMAFSTAFTTLLAMLLASGDPRMAQLLTWISGSTYGVD  521
Xm 439  TSGNVFVWLLPAGS-LGAALTLLLILFISARQRFSAQHMLLTGIAISTVFGTFVALVLASGDPRTGVLLAWLSGSTYGVE  517
Pf 464  FGDAGHTARIAASA-AGALAALLAILWFSQRSRFAPDRVLLAGVAIGALFQAVVAVAIASGGERATTLLQWLAGSTYTIT  542
Ps 442  IAEPTYGH-RMFGGFSGAAIVL-TVLFAMSRKVRSGNQFLIIGVSLGALLSALISVILASGDPRAVSLISWMAGSTYGIS  519
Vr 442  GVGASVVT-LSIGGLVGALLTVLIIVLLNRKSGFQPERILLTGIAITALMNAIQSFILATGDPRSYQALAWLSGSTYYVS  520
Od 457  F-GVSAVW-IPIGAMIGSFVFFVIVMALAIRAQFQPTILALLGIGVSAFGSAIIQILVVQADLGVSSALTWLSGTTYARG  534
Sa 458  --SLSAAF-IPLGAFMGALAAFLVVMGLSYRAGFQPSLLALLGIGVSAFGSALIQIMVVQADMAVASALTWLSGSTYAKS  534

              TM16                                 TM17                 TM18
521  DAQVWRTGIV-MVILLAITPLCRRWLTILPLGGDTARAVGMALTPTRIALLLLAACLTATATMTIGPLSFVGLMAPHIAR  599
521  GGQVTRTAIV-MVILLAIVPLCRRWLTILPLGGDAARAVGMALTPSRIALLALAACLTATATMTIGPLSFVGLMAPHIAR  599
522  GQHAVYSFIV-MAVLLALTPLCRRWLTVLPLGGDTARAVGLALTPSRVTLLMLASGLTAAATMTIGPLSFVGLMAPHITR  600
522  APQALRTALV-AVVLVCIAPFASRWLTILPLGSATARAVGMALTPVRLSLLLLAAALTAAATLTIGPLSFVGLMAPHMAR  600
518  PAQALFTLVI-ALVLIALTPLYRRWLTILPLGSINAQAVGISLIRSRFLILLIASALTASATLMTGPLSFVGLMAPHMAR  596
543  PTDAALALVL-CVGLCAATPLFNRWLQILPLGEASARALGVSTRGARFALLLLIALLTAAATLVVGPLSFVGLTAPHFAR  621
520  RQIAWISLAF-AVSGLAAVTLFLRPLQQFALGDISAHSHGVDVAKFRVMILGFAALLTAIATLVVGPLSFVGLMAPHMAQ  598
521  MDLLLPLALS-AFVFIGLSFVCVRWLDILPIGASSSKALGIKVDRSRAILLLLVACLTVSATLVVGPISFVGLLAPHLAR  599
535  WSELFNYLIWPAFILLPILFLKIRVMDTLALGDDTAKGLGLKVMSTRFQLAFLATVLASFSVAAVGAIGFVGLIAPHFSR  614
535  WEALIDYLLWPMVILVPILYLQSKNLNVLSLGDETAKGLGLRVTRTRFEMALVASLLAAASVAAVGTISFVGLIAPHVAR  614

                                                                               Identities
                 TM19                     TM20
600  MMGFRRTMPHIVISALVGGLLLVFADWCGRMVLFPFQIPAGLLSTFIGAPYFIYLLRKQSR-----  660  660/660 (100%)
600  MLGFRRTIPHMTISALAGGVLLVIADWCGRMALFPYQIPAGLLSSFIGAPYFIYLLRKQSR-----  660  586/660 (88.8%)
601  MMGFRRALPQLVISALLGGLLLVVADWCGRMVMFPYQIPAGLLSTFIGAPYFVYLLRKQSR-----  661  533/662 (80.5%)
601  MLGFRRALPQLAVAALLGGGLMLVADWCGRMAAFPNQIPAGLLATFIGAPYFVWLLRKAT------  660  462/659 (70.0%)
597  MMGFRKAMPQLMIGMLLGGLLMLLADWFGRMLLFPYQIPAGLLAAFIGVPYFIVLLRRQAK-----  657  383/636 (60.2%)
622  LLGARRPLQQVLLAAPIGALTIVVADWLGRALLMPRELPAGLVATLIGAPYLMWLLGRRR------  681  318/640 (49.7%)
599  RLGLTRGLAHLLGSALLGALLMAIADFIGRTIYFPWQLPTGLVATLLGGPVFALLLIRTGRRSMQH  664  270/664 (40.7%)
600  LFGFNKASQHMLCAALLGAGVMLLADWLGRQVLYPQEIPAGLMASLIGGLYLMWGLRRL-------  658  240/659 (36.4%)
615  LLVGPANQRLLPVTALIGGLLLVVADLFSRVLLAPNEIPTGIIVAIIGAPYFLWLMKKRA------  674  218/666 (32.6%)
615  MFLGPGHKYLLPCSALLGAFFLVVADILSRTLLVPKEIPSGILVALMGAPYFLWLMYKSSLPKVN-  679  226/653 (34.6%)
```

Supplementary Figure 13. Association between FhuC and FhuB. a, Along the central channel in FhuB, the FhuCDB complex is divided into two halves and superimposed together with N-half multi-colored and C-half in gray. **b,** Zoom in view of the FhuC-FhuB interface mediated by the coupling helices. Conserved residue FhuB-G226 (equivalent to FhuB-G559) is shown in ball mode, and the surface representation of FhuC is shown in green.

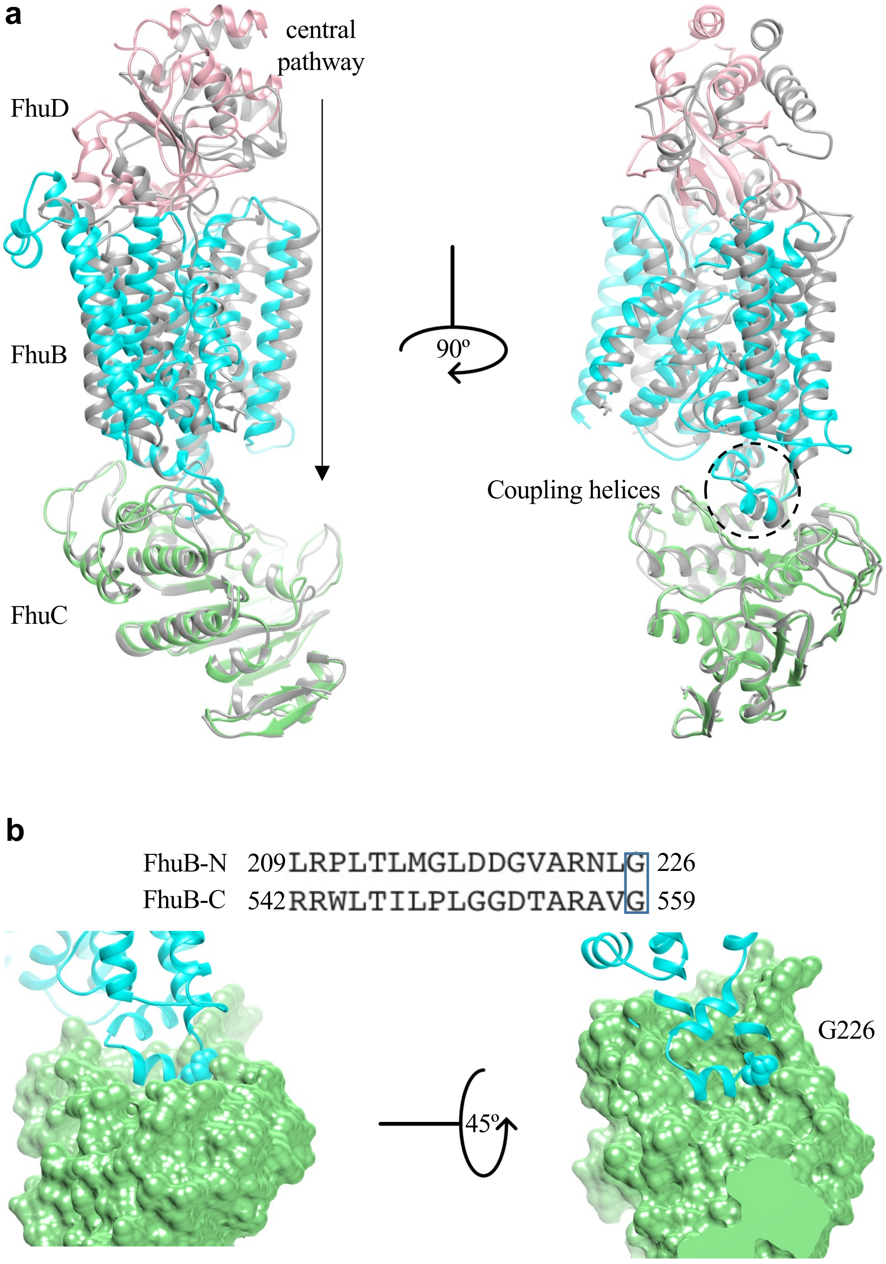

Supplementary Figure 14. MST analysis of the binding between FhuCB and FhuD. **a**, FhuCB and FhuD. **b**, FhuCB and FhuD with ATP-$MgCl_2$. **c**, FhuCB and FhuD with ferrichrome. **d,** FhuCB and FhuD with ferrichrome and ATP-$MgCl_2$.

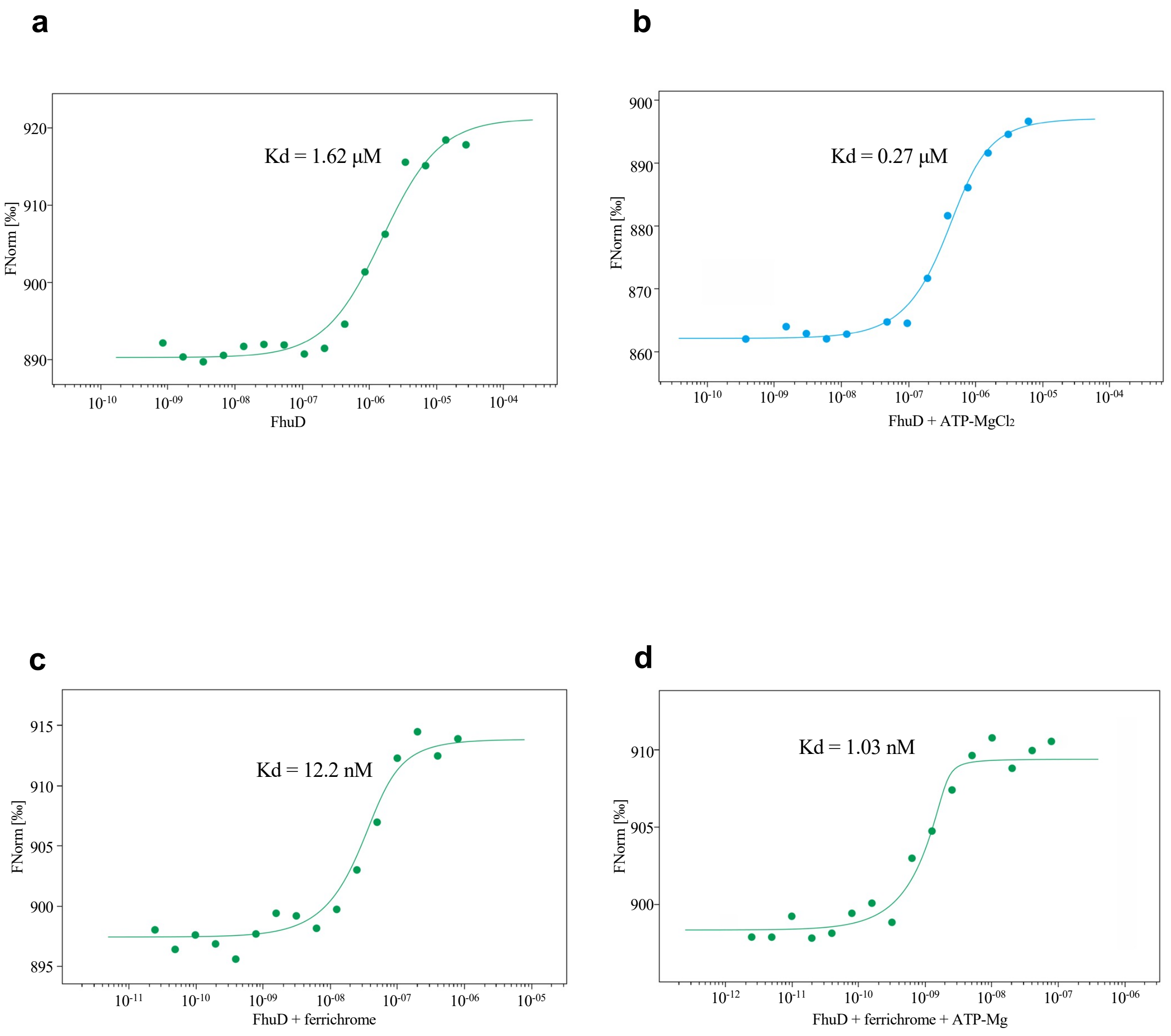

Supplementary Figure 15. Comparison between FhuB and BhuU both in the inward-open conformation. FhuCDB is superimposed with BhuUVT (PDB:5B58, Naoe et al., 20). Looking down the translocation pathway from the periplasmic side, the sliced view of the TMs shows a narrower channel in FhuB (cyan) than BhuU (gray) because of the substantial movement of TM15.

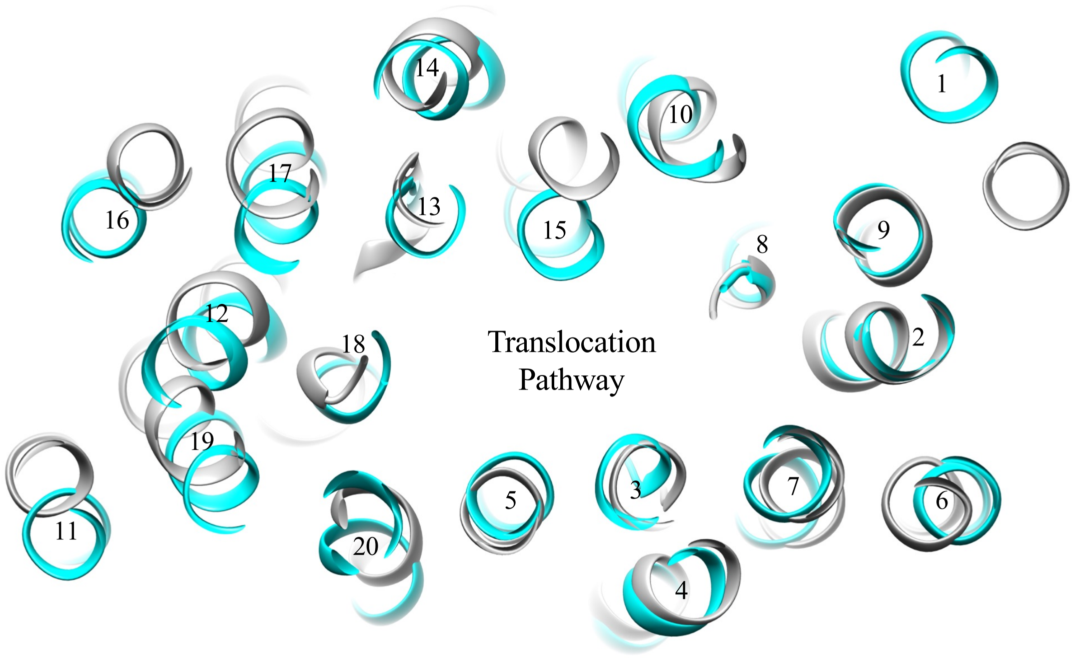

Supplementary Figure 16. Methionine residues found in the structure of yersiniabactin importer YbtPQ (PDB:6P6J) around the central translocation pathway. YbtP is in dark blue, YbtQ is in light blue, the substrate Fe^{3+}-yersiniabactin is in gray, Met residues are shown in stick mode and colored by heteroatom. The dotted line shows a putative translocation pathway.

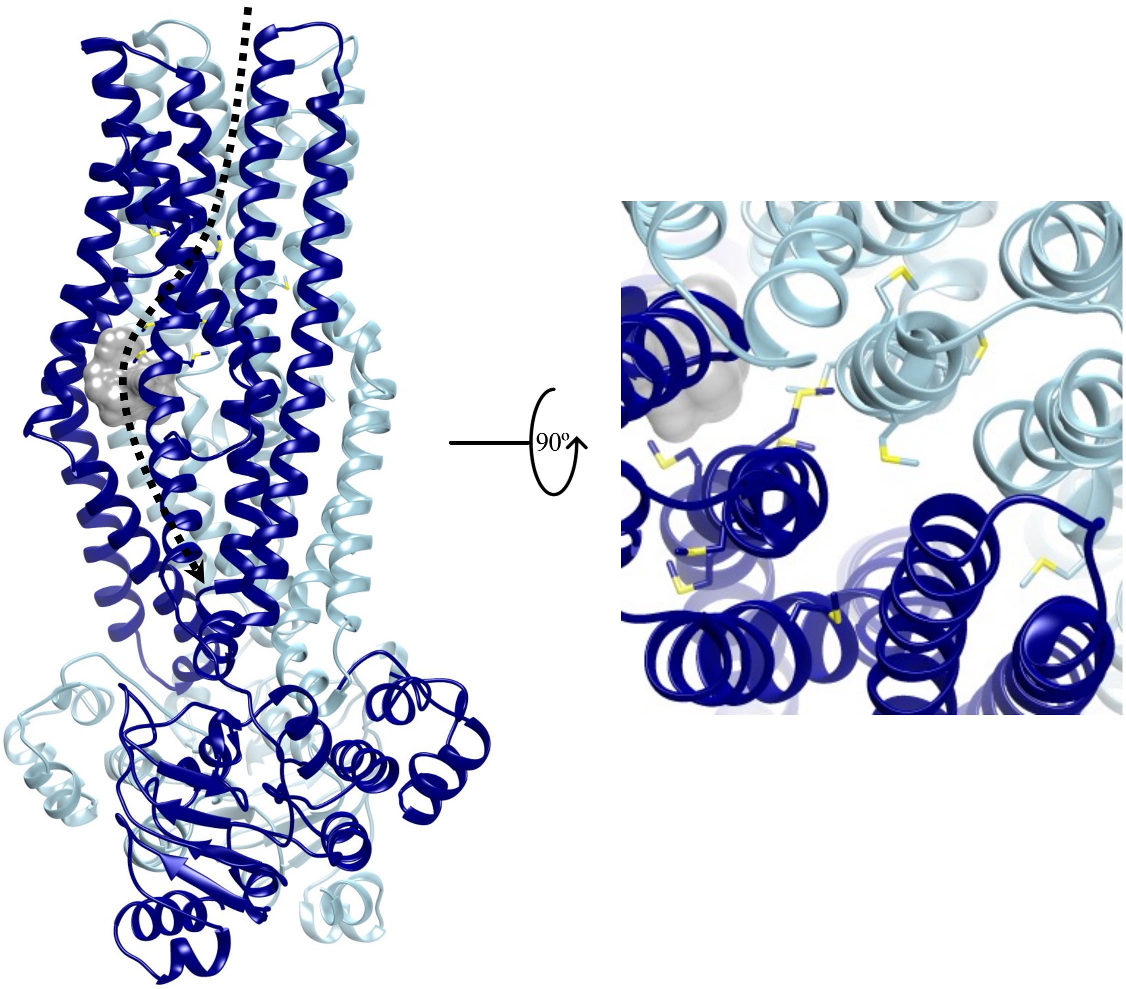

Supplementary Figure 17. Substrate transport efficiency of FhuCB with Met mutations. a, Comparison of the amount of FhuCB reconstituted into liposomes. Lane 1: wild-type; lane 2: Met to Ala mutant. **b,** Liposomes with Met mutations in FhuB show reduced transport efficiency comparing to wild-type. **c,** Met mutations in FhuB do not change ATPase activity comparing to wild-type. Error bars are the standard deviation from n=3 independent measurements.

a

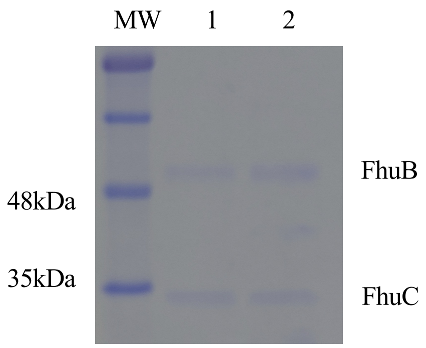

b

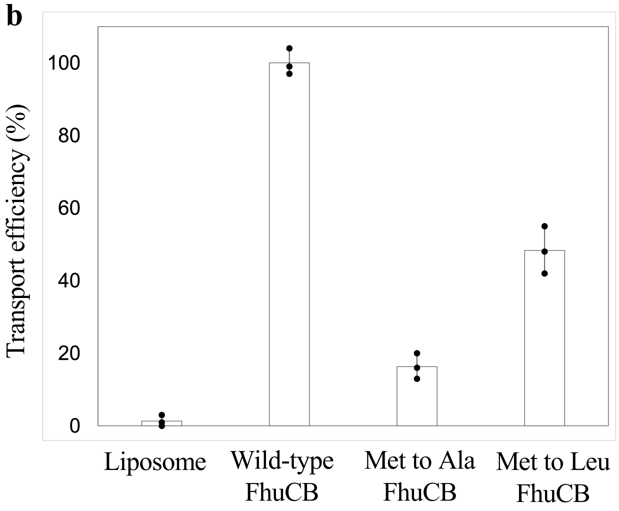

c

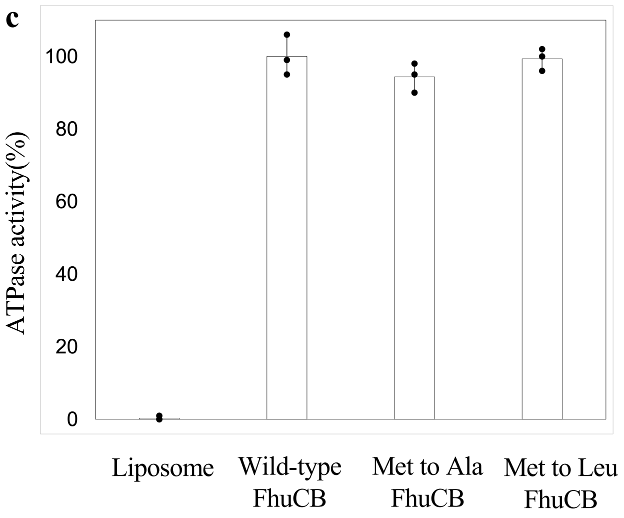

Supplementary Figure 18. Comparison between FhuCDB and BtuCD structures. a, Closed cytoplasmic gate I (TM5 and its equivalent) in BtuCDF (2QI9, gray) is open in FhuB (cyan). **b**, Closed cytoplasmic gate II (L2 and its equivalent) in the outward-open BtuCD (4R9U, gray) is also open in FhuB (cyan).

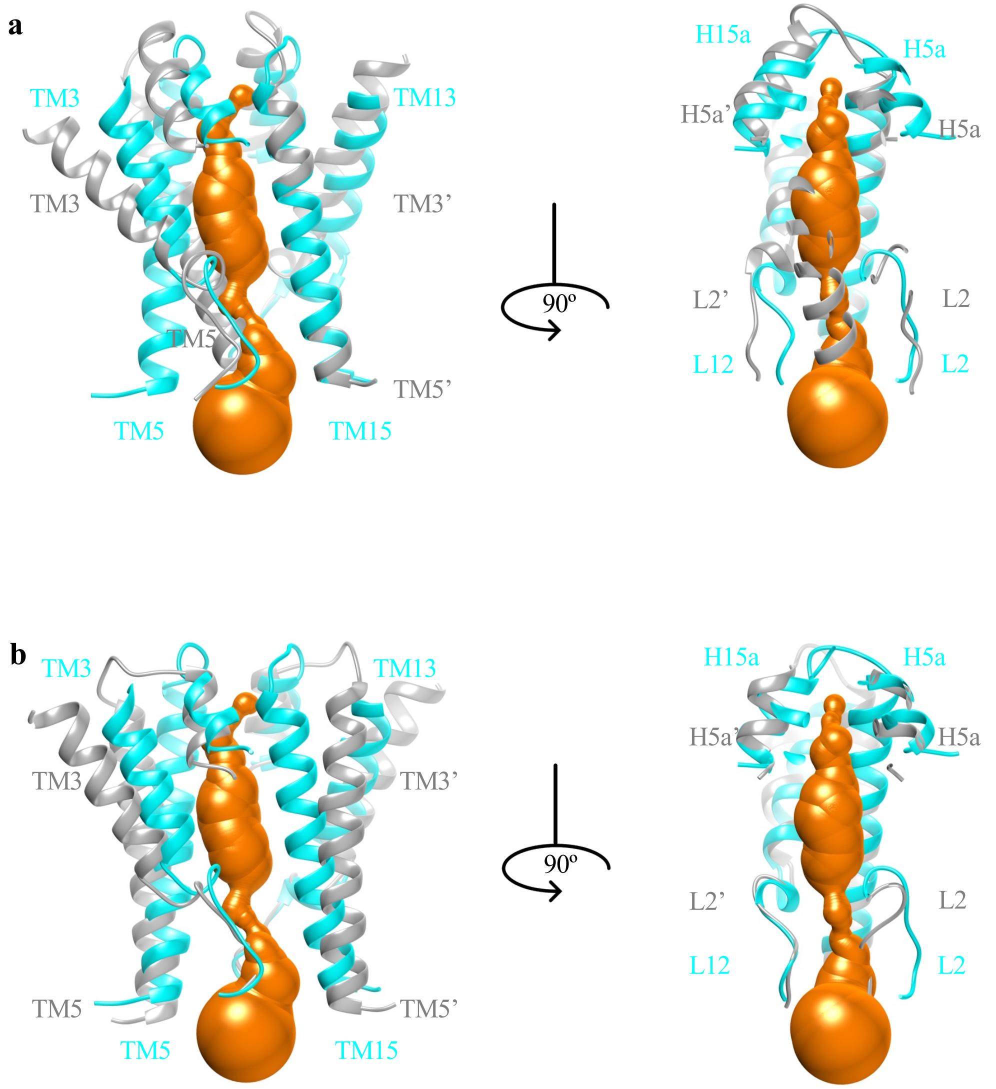

Supplementary Figure 19. Uncropped gels for supplementary figures 2b, 3b and 17a.

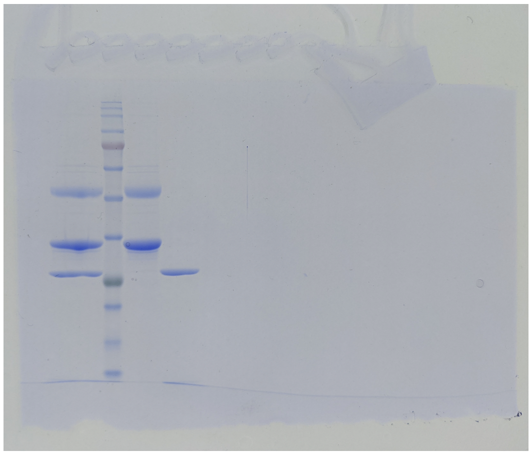

Uncropped supp fig 2b

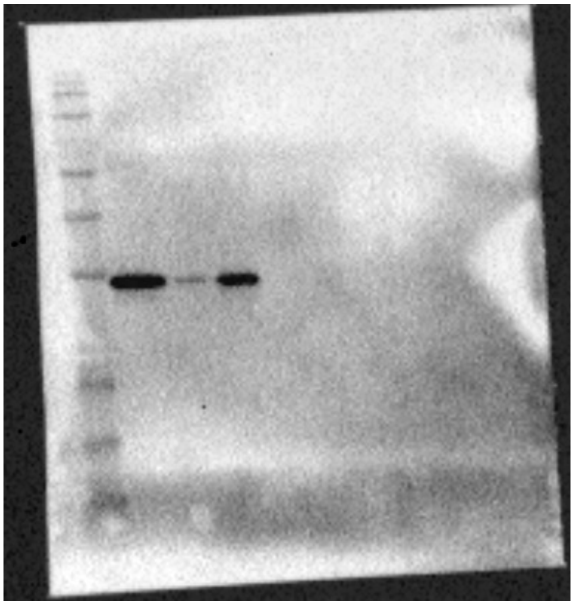

Uncropped supp fig 3b

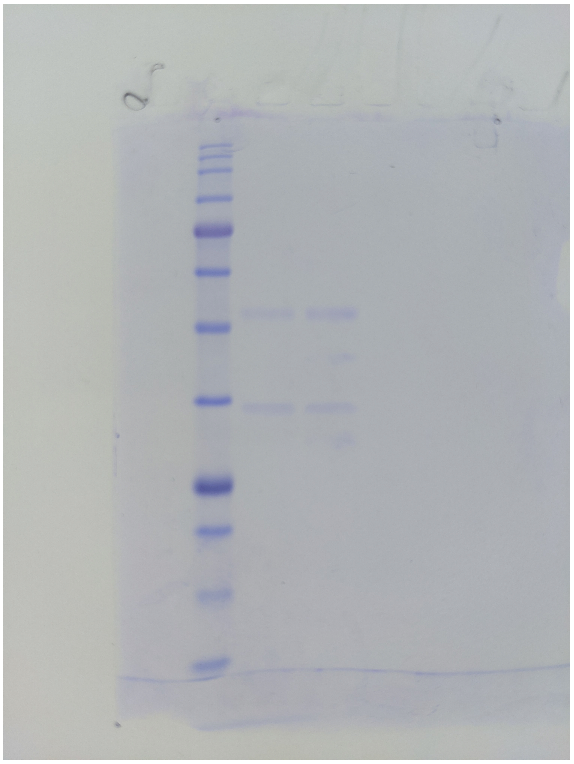

Uncropped supp fig 17a

Supplementary Table 1: Kinetic constants of ATPase activity from known Type II importers.

Type II importer	Substrate	K_m (mM)	V_{max} (nmol/mg/min)	N_{hill}	Environment	Reference
BtuCD	Vitamin B12	<0.05	200~1200		Proteoliposome, LDAO, DDM, FOS12, Triton X-100	(Borths et al., 2005)
		0.017	1090	1.03	LDAO	(Tal et al., 2013)
		0.02	360	2.09	proteoliposome	
HmuUV	Heme	0.18	173	1.18	DDM	(Woo et al., 2012)
BhuUV	Heme	0.028	5000		DM	(Naoe et al., 2016)
hiMolBC	Molybdate Tungstate	0.039	3680		proteoliposome	(Vigonsky et al., 2013)
afModBC	Molybdate Tungstate		25		C12E8	(Hollenstein et al., 2007)
FhuCB	Ferrichrome	0.69	1160	1.93	proteoliposome	This study

Borths, E.L., Poolman, B., Hvorup, R.N., Locher, K.P., and Rees, D.C. (2005). In vitro functional characterization of BtuCD-F, the Escherichia coli ABC transporter for vitamin B12 uptake. Biochemistry *44*, 16301-16309.

Hollenstein, K., Frei, D.C., and Locher, K.P. (2007). Structure of an ABC transporter in complex with its binding protein. Nature *446*, 213-216.

Naoe, Y., Nakamura, N., Doi, A., Sawabe, M., Nakamura, H., Shiro, Y., and Sugimoto, H. (2016). Crystal structure of bacterial haem importer complex in the inward-facing conformation. Nat Commun *7*, 13411.

Tal, N., Ovcharenko, E., and Lewinson, O. (2013). A single intact ATPase site of the ABC transporter BtuCD drives 5% transport activity yet supports full in vivo vitamin B12 utilization. Proc Natl Acad Sci U S A *110*, 5434-5439.

Vigonsky, E., Ovcharenko, E., and Lewinson, O. (2013). Two molybdate/tungstate ABC transporters that interact very differently with their substrate binding proteins. Proc Natl Acad Sci U S A *110*, 5440-5445.

Woo, J.S., Zeltina, A., Goetz, B.A., and Locher, K.P. (2012). X-ray structure of the Yersinia pestis heme transporter HmuUV. Nat Struct Mol Biol *19*, 1310-1315.

Supplementary Table 2: Cryo-EM data collection, refinement and validation statistics

	FhuCDB in LMNG
**Data Collection and Processing**	
Microscope	Titan Krios
Voltage (kV)	300
Magnification (nominal)	92,000
Electron Dose ($e^-/Å^2$)	65
Camera	Gatan K3
Defocus range (um)	-1 ~ -2.5
Pixel size (Å)	0.399 (super resolution)
Movies collected	9142
Symmetry imposed	C1
Final particle images (no.)	128,131
Map resolution (Å)	3.4
FSC cutoff	0.143
Map resolution range	3.2 ~ 6.5
Sharpening B-factor ($Å^2$)	-106.7
Software used to process data	cryoSPARC, RELION 3.1
**Refinement statistics**	
Initial model used	1ESZ
Model resolution (FSC = 0.5)	3.6
Correlation Coefficient (Mask)	0.76
Number of protein atoms (non-H)<br>Residues	10469<br>1405
R.m.s. deviations	
Bonds (Å)	0.005
Bond angles (º)	0.715
Validation	
MolProbity score	1.91
Clash score	10.40
Poor rotamers (%)	0.00
Ramachandran plot	
Favored (%)	94.62
Allowed (%)	5.38
Disallowed (%)	0
EMDB access code	EMD-23251
PDB access code	7LB8

Supplementary Table 3: Interactions between FhuCB (wild-type and FhuB mutants) and FhuD analyzed using MST. All measured K_d values between FhuCB and FhuD are listed.

	FhuD	FhuD + ATP-Mg	FhuD-ferrichrome	FhuD-ferrichrome + ATP-Mg
FhuCB wild-type	1.62 µM	0.27 µM	12.2 nM	1.03 nM
R390A				75.8 nM
Q636A				90.2 nM
Q507A, T510A				79.2 nM
S515A, Y517A				Not detectable
D170A, Q171A				Not detectable
S57A				88.4 nM
E304A				15.1 nM
T182A, T184A				Not detectable
