# Supplementary material for: Cryo-EM reveals unique structural features of the FhuCDB Escherichia coli ferrichrome importer
Source: Commun Biol. 2021 Dec 9;4:1383. doi: 10.1038/s42003-021-02916-2 (PMC8660799; doi:10.1038/s42003-021-02916-2)
Supplement: Supplementary file 2 — Description of Additional Supplementary Files [file 42003_2021_2916_MOESM2_ESM.pdf]

Description of Additional Supplementary Files

File name: Supplementary Data 1.
Description: Data used to generate curves in Figure 1.
